# The resealing factor S100A11 interacts with annexins and extended synaptotagmin-1 in the course of plasma membrane wound repair

**DOI:** 10.3389/fcell.2022.968164

**Published:** 2022-09-19

**Authors:** Arsila P. K. Ashraf, Volker Gerke

**Affiliations:** Institute of Medical Biochemistry, Centre for Molecular Biology of Inflammation, University of Münster, Münster, Germany

**Keywords:** calcium-binding proteins, cell damage, plasma membrane resealing, extended synaptotagmin, laser ablation

## Abstract

After damage, cells repair their plasma membrane in an active process that is driven by Ca^2+^ entering through the wound. This triggers a range of Ca^2+^-regulated events such as the translocation of different Ca^2+^-binding proteins to the wound site which likely function in the repair process. The translocated proteins include Ca^2+^/phospholipid binding proteins of the annexin (ANX) family and S100A11, an EF hand-type Ca^2+^-binding protein which can interact with ANX. The molecular mechanism by which S100A11 mediates PM wound repair remains poorly understood although it likely involves interactions with ANX. Here, using S100A11 knockout endothelial cells and expression of S100A11 mutants, we show that endothelial S100A11 is essential for efficient plasma membrane wound repair and engages in Ca^2+^-dependent interactions with ANXA1 and ANXA2 through its C-terminal extension (residues 93–105). ANXA2 but not ANXA1 translocation to the wound is substantially inhibited in the absence of S100A11; however, the repair defect in S100A11 knockout cells is rescued by ectopic expression of an ANX interaction-defective S100A11 mutant, suggesting an ANX-independent role of S100A11 in membrane wound repair. In search for other interaction partners that could mediate this action of S100A11 we identify extended synaptotagmin 1 (E-Syt1), a protein tether that regulates endoplasmic reticulum-plasma membrane contact sites. E-Syt1 binds to S100A11 in the presence of Ca^2+^ and depletion of E-Syt1 interferes with wound site recruitment of S100A11 and proper membrane resealing. Thus, the role of S100A11 in membrane wound repair does not exclusively dependent on ANX interactions and a Ca^2+^-regulated S100A11-E-Syt1 complex acts as a yet unrecognized component of the membrane resealing machinery.

## Introduction

Many cells display the ability to repair their membrane when damaged during the ordinary wear and tear of normal physiology or as a result of pathogenesis or injury. Repair is achieved by two complementary processes: first, immediate membrane resealing to stop the loss of cytoplasm and the influx of toxic components and then, rebuilding of cellular structures that were damaged or lost (Cooper and McNeil, 2015). The latter process is characterized by restoration of the cortical cytoskeleton (Sonnemann and Bement, 2011) and a reconstitution of the normal (resting) plasma membrane (PM) lipid distribution (Vaughan et al., 2014; Davenport et al., 2016; Ashraf and Gerke, 2021). Cell membrane repair is increasingly recognized to have important implications for human health as excessive cell damage and/or defective repair results in or is associated with a variety of pathologies, including cardiomyopathy, muscular dystrophies, diabetic myopathy and neurodegenerative diseases (reviewed in Ammendolia et al., 2021; Dias and Nylandsted, 2021).

PM wounds are frequently suffered by cells of respiratory and gastrointestinal tracts, skeletal muscle, heart and vascular system, which are subject to mechanical stress *in vivo* (McNeil and Ito, 1989; McNeil and Khakee, 1992; McNeil, 1993). To survive and maintain membrane integrity, several cell types have been shown to be capable to actively reseal their PM wounds in a cell-autonomous process. PM damage leads to disruption of ion homeostasis which involves rapid (within ms) and pathological Ca^2+^ influx into the injured cell. The resulting cytosolic Ca^2+^ rise is utilized by the cell to sense the damage and initiate the repair process facilitated by Ca^2+^-mediated disintegration of the cortical cytoskeleton. Ca^2+^ also recruits a cohort of proteins that limit wound expansion and support resealing (McNeil and Kirchhausen, 2005; Cooper and McNeil, 2015). Most likely these proteins participate in mediating membrane fusion processes such as endocytosis, exocytosis or membrane shedding that have been proposed to assist membrane resealing (Blazek et al., 2015; Jimenez and Perez, 2017). In cooperation with membrane fusion events, cytoskeletal structures have also been described to support resealing by wound constriction. Thus, cell membrane wound repair most likely involves several parallel and overlapping processes working in concert.

Several Ca^2+^-binding proteins such as annexins, dysferlin and synaptotagmins translocate to sites of PM injury and actively support resealing, among other things by recruiting different intermediary effectors. Annexins (ANX) (for review see, Koerdt et al., 2019) contain a unique Ca^2+^-sensing and membrane binding core domain comprising four so-called ANX repeats, each with five α-helices separated by loops that coordinate Ca^2+^ ions (Gerke and Moss, 2002). Through membrane binding and interactions with other proteins, ANX act as critical mediators of the emergency repair response, for example by inducing vesicle aggregation and membrane fusion (Blackwood and Ernst, 1990) and by the formation of ordered 2D arrays and membrane curvature that prevent wound expansion and prepare the membrane for resealing (Bouter et al., 2011; Miyagi et al., 2016; Boye et al., 2017, 2018; Hakobyan et al., 2017; Lin et al., 2020).

Some ANX can interact with members of the S100 family of EF hand Ca^2+^-binding motif-containing proteins which also sense increases in cytosolic Ca^2+^ levels (Heizmann and Fritz, 2010). S100 proteins are composed of two consecutive EF hands that are separated by a linker sequence and followed by a C-terminal extension that is unique to each member of the family. They exist as non-covalent symmetric homo- or hetero-dimers and respond to elevated Ca^2+^ levels by conformational changes which expose hydrophobic residues that bind target sequences such as the N-terminal regions of some ANX (reviewed in Rintala-Dempsey et al., 2008). The two best-characterized S100-ANX complexes are S100A10-ANXA2 (Réty et al., 1999; Lewit-Bentley et al., 2000) and S100A11-ANXA1 (Mailliard et al., 1996), each forming heterotetrameric units that allow two membrane-bound ANX to be linked via an S100 dimer thereby facilitating close apposition of adjacent membranes, for example in the course of membrane aggregation and fusion during the resealing process.

S100A11 has also been shown to associate with ANXA2 and this interaction is required for efficient PM wound repair and subsequent survival of metastatic cancer cells (Jaiswal et al., 2014). A Ca^2+^-dependent translocation of S100A11 to the site of PM injury has also been described in endothelial cells where both, S100A11 and ANXA2 serve as positive regulators of the resealing process (Koerdt and Gerke, 2017; Ashraf and Gerke, 2021). However, the mechanisms by which S100A11 is recruited to membrane ruptures and facilitates PM wound repair are still unresolved.

Here, we show that loss of S100A11 results in defective membrane repair in endothelial cells and that endothelial S100A11 interacts with ANXA1 and ANXA2 at elevated Ca^2+^ levels in a manner requiring residues in the unique C-terminal extension. Loss of S100A11 is associated with abnormal trafficking of ANXA2 to the wound, but S100A11 translocation and S100A11-mediated repair of endothelial membrane wounds are not dependent on ANX binding. Rather, they appear to depend on the interaction of S100A11 with a novel binding target described here, the extended synaptotagmin-1 (E-Syt1), a protein localized to endoplasmic reticulum-plasma membrane (ER-PM) contact sites.

## Results

### S100A11-deficient endothelial cells are defective in plasma membrane repair

To induce localized plasma membrane (PM) wounds in endothelial cells at the single-cell level, we used an established laser ablation protocol, which involves rupturing the cell membrane with an intense laser pulse (Koerdt and Gerke, 2017). As we had shown that this ablation-based wounding and the resulting influx of extracellular Ca^2+^-triggered a rapid translocation of S100A11 towards the injury sites (Koerdt and Gerke, 2017; Ashraf and Gerke, 2021), we sought to determine if S100A11 is functionally involved in PM wound repair in endothelial cells by fully abolishing the expression of endogenous S100A11. For this purpose, we employed genome editing by the CRISPR/Cas system (Supplementary Figure S1A) to generate stable S100A11 knockouts in the endothelial EA.hy926 cells (S100A11-KO). EA.hy926 is a fusion cell line of HUVEC and A549 epithelial cells which preserves the characteristics of vascular endothelial cells (Edgell et al., 1983). EA.hy926 cells were used instead of HUVEC, as the latter being primary cells were unsuitable for long-term maintenance. For six different clones generated, KOs were verified by immunoblotting. All clones displayed a lack of S100A11 expression, in contrast to EA.hy926 wild type (WT) cells (Supplementary Figure S1B). Two clonal populations (#1 and #3) were selected for further analysis.

**FIGURE 1:**
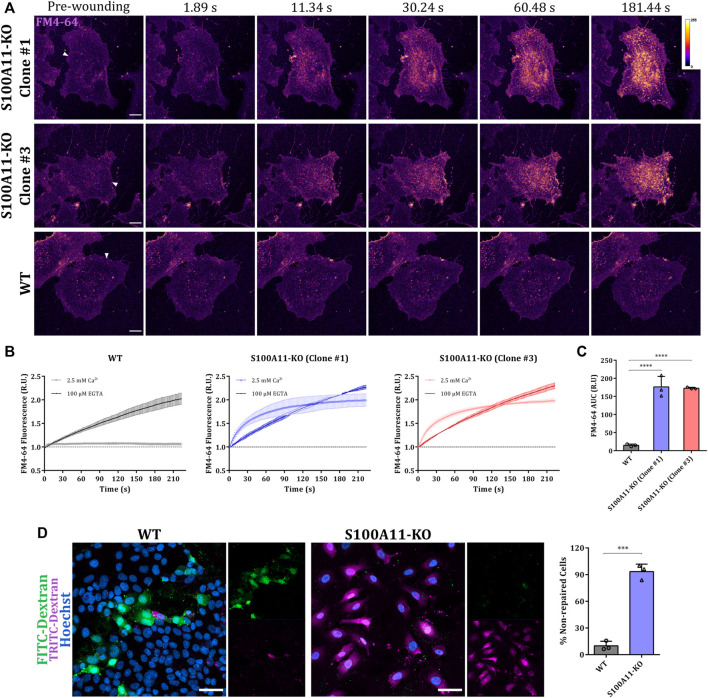
Plasma membrane repair is defective in S100A11-KO EA.hy926 cells. **(A)** Representative images of FM4-64 influx following membrane damage in S100A11-KO, clone #1 and #3, or WT EA.hy926 cells (see Supplementary videos S1, S3). Cells kept in Tyrode’s buffer supplemented with 2.5 mM Ca^2+^ and 5 μg/ml FM4-64 were injured by laser ablation at 820 nm (near infrared) directed at the plasma membrane on the lateral edge (white triangles represent the wound sites). Wounding occurred at t = 0; pre-wounding and post-wounding time points are shown. Calibration bar of fluorescence intensity is provided in the upper right panel. Scale bars = 10 µm. **(B)** Time course of whole-cell FM4-64 fluorescence normalized to fluorescence before injury in WT (left) or S100A11-KO cells (clone #1: middle and clone #3: right) laser-ablated in the presence of extracellular Ca^2+^ or EGTA. **(C)** AUC values of FM4-64 fluorescence in WT or S100A11-KO cells damaged in the presence of extracellular Ca^2+^. S100A11-KO, *n* = 27 cells per clone; WT, *n* = 18 cells. Results were pooled from three independent experiments. Statistical analysis was performed with ordinary one-way ANOVA. **(D)** Exemplary fields of mechanically wounded WT or S100A11-KO cells stained with FITC-Dextran, TRITC-Dextran and Hoechst. The protocol (see Materials and Methods) permits the distinction between wounded and repaired cells (that had taken up only FITC-Dextran) and wounded but non-repaired cells (that had taken up FITC-Dextran and TRITC-Dextran). Hoechst was included as a marker for all cells. Scale bars = 50 μm. Quantification of non-repaired WT or S100A11-KO cells, represented as a percentage of total wounded cells (right). Results were pooled from three independent experiments (*n* = 75 fields). Statistical analysis was carried with two-tailed Student’s t test. Data are mean ± SD.

Next, we evaluated the competence of WT or S100A11-KO cells (from two different clonal populations, #1 and #3) to repair laser-induced membrane wounds in the presence or absence of extracellular Ca^2+^ (Figure 1A and Supplementary Figure S2). PM wound repair efficiencies of injured cells were evaluated by the cell-impermeant styryl dye FM4-64 which fluoresces intensely when inserted into lipid-rich membranes (Betz et al., 1996). Upon membrane rupture, FM4-64 enters the cell, labels internal membranes and produces a bright fluorescence which is restricted to the wound site when PM resealing occurs but penetrates the entire cell when repair is defective (Ashraf et al., 2021). As expected, FM4-64 influx was continuous over time in laser-ablated WT or S100A11-KO EA.hy926 cells kept in an EGTA (Ca^2+^ chelator) containing medium (Supplementary Figure S2 and Figure 1B), revealing that PM resealing in EA.hy926 cells depends on the influx of extracellular Ca^2+^ as shown previously for other mammalian cell types including HUVEC (Detrait et al., 2000; Togo et al., 2000; McNeil et al., 2006; Jaiswal et al., 2014; Carmeille et al., 2015; Koerdt and Gerke, 2017). In the presence of extracellular Ca^2+^, while FM4-64 fluorescence in laser-ablated WT cells was restricted to the immediate vicinity of the wound, the fluorescence increase was more rapid and sustained in injured S100A11-KO cells (Figures 1A,B). To quantitatively compare the responses of WT or S100A11-KO cells to damage, we calculated the area under the curve (AUC) for FM4-64 influx dynamics. S100A11-KO FM4-64 AUCs were significantly greater than those of WT EA.hy926 cells (Figure 1C). As both clonal populations (#1 and #3) showed indistinguishable phenotypes in the assays described below, results are only displayed for one of the two populations (#1). We also employed a mechanical wounding assay based on dual-labeled dextran probes, FITC-Dextran and TRITC-Dextran which stain repaired and non-repaired cells, respectively (Supplementary Figure S3; Defour et al., 2014). Figure 1D shows that S100A11-KO cells are also significantly impaired in their ability to repair glass beads-induced mechanical wounds as opposed to WT cells.

### Ectopically expressed S100A11 traffics to the wound site and rescues the membrane repair defect

**FIGURE 2:**
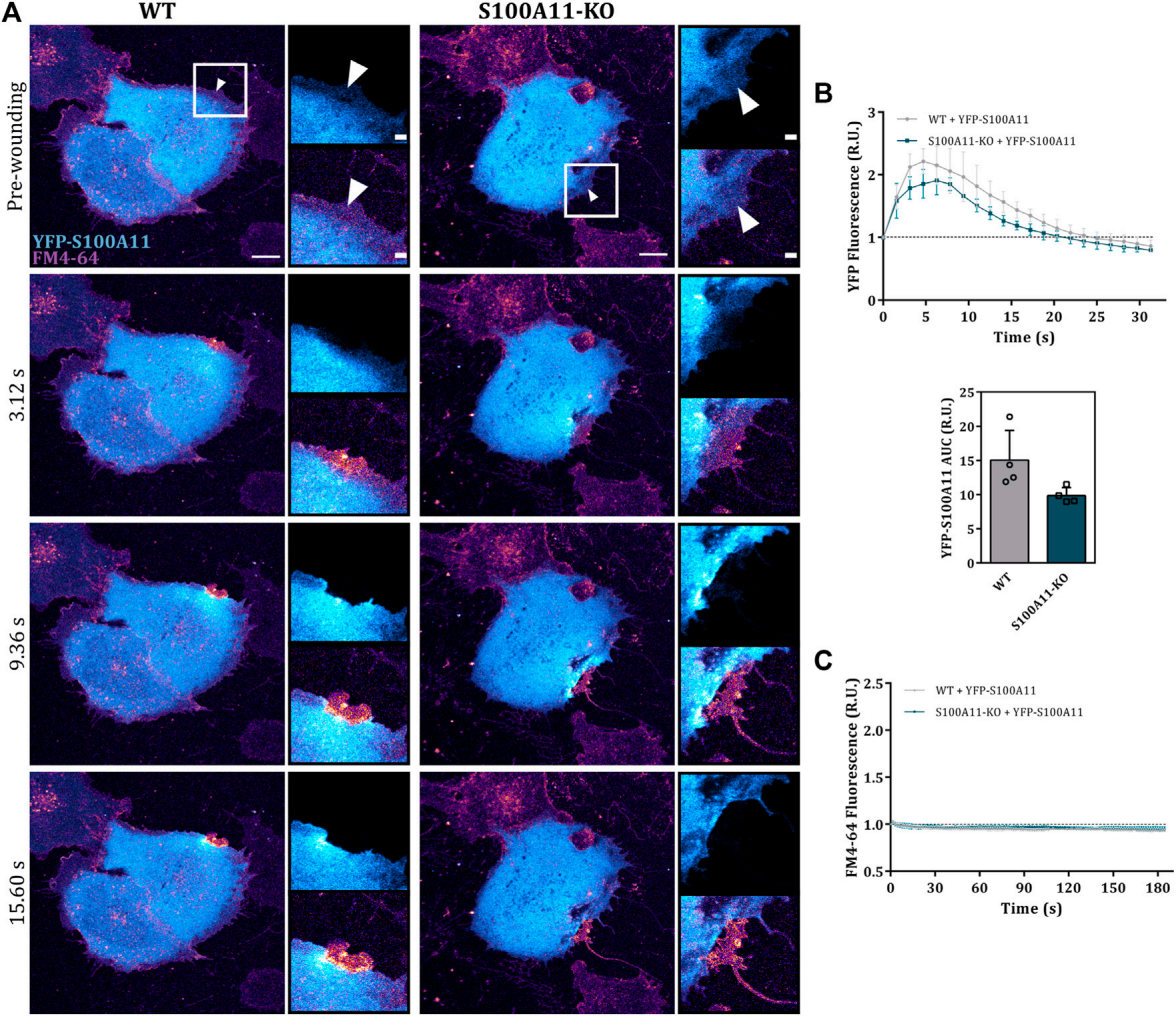
S100A11 translocates to the injury site in response to wounding. **(A)** Time-lapse images of wave-like S100A11 translocation to laser-induced plasma membrane wounds in WT or S100A11-KO cells, respectively (Supplementary Video S5). YFP-S100A11 transfected cells kept in Tyrode’s buffer supplemented with 2.5 mM Ca^2+^ and 5 μg/ml FM4-64 were injured at the lateral membrane edge (white triangles represent the wound sites). Wounding occurred at t = 0; pre-wounding and post-wounding time points are shown. Scale bars = 10 µm. Insets highlight the immediate vicinity of the wound. Scale bars = 2 μm. **(B)** Time course of YFP-S100A11 fluorescence in WT or S100A11-KO cells, normalized to fluorescence before injury. Mean fluorescent intensity was measured in the area next to the wound site (a circular ROI of 10 μm around the wound) for each acquisition time point. YFP-S100A11 AUCs for WT and S100A11-KO cells are also shown. Results were pooled from four independent experiments (*n* = 24). Statistical analysis was performed with two-tailed Student’s t test. **(C)** Time course of whole-cell FM4-64 fluorescence in WT or S100A11-KO cells ectopically expressing YFP-S100A11, normalized to fluorescence before injury. Control experiments carried out in the absence of extracellular Ca^2+^ (EGTA containing medium) showed no resealing under the experimental condition used here, indicative of a still Ca^2+^-dependent repair process. Statistical comparison between FM4-64 AUCs was performed with two-tailed Student’s t test. Data are mean ± SD.

Given that endothelial cells lacking S100A11 showed a PM resealing defect, we next analyzed whether and how S100A11 is recruited towards the site of injury in EA.hy926 cells by expressing fluorescently tagged S100A11 (YFP-S100A11) in WT or S100A11-KO cells. We observed an immediate translocation of S100A11 towards the site of damage in WT as well as S100A11-KO cells (Figure 2A). This occurred in a wave-like fashion similar to what was reported previously in HUVEC (Koerdt and Gerke, 2017). Some static but short-lived punctae of unknown origin appeared in the S100A11 wavefront (Figure 2A; insets). To quantify S100A11 recruitment kinetics in wounded cells, the YFP fluorescence measured in a defined area around the wound (Supplementary Figure S4) at a given time point was divided by pre-wounding fluorescence and plotted versus time. In WT cells, S100A11 was recruited rapidly and fluorescence around the wound returned to baseline levels within 20–25 s post-wounding (Figures 2A,B). A similar kinetics and comparable AUC values were observed for S100A11-KO cells (Figure 2B), indicating that the machinery necessary for S100A11 trafficking is not affected. Importantly, re-expression of YFP-S100A11 in S100A11-KO cells rescued the repair defect as revealed by the significantly reduced FM4-64 influx (Figures 2A,C).

### Ca^2+^-activated S100A11 interacts with ANXA1 and ANXA2 *via* its C-terminal domain

Wound-directed S100A11 trafficking in the form of a dynamic wave resembles that reported for annexins (ANX) (Koerdt and Gerke, 2017; Ashraf and Gerke, 2021) which are thought to show this type of recruitment based on their Ca^2+^-dependent phospholipid binding capability. However, unlike ANX, S100A11 lacks direct membrane binding properties, but is described to form Ca^2+^-mediated heterotetrameric complexes with annexins, namely ANXA1 (Réty et al., 2000; Rintala-Dempsey et al., 2008), ANXA2 (Rintala-Dempsey et al., 2006; Streicher et al., 2009) and ANXA6 (Chang et al., 2007; Rintala-Dempsey et al., 2008). As S100A11-ANXA2 complexes have been implicated in PM wound repair of cancer cells (Jaiswal et al., 2014), we hypothesized that in the course of endothelial PM wound repair, S100A11 could engage in Ca^2+^-dependent interactions with ANX and its recruitment to PM wounds might be ANX dependent and therefore occurs in a wave-like manner. To screen for potential interactions between S100A11 and ANX in endothelial cells, lysates from HUVEC expressing GFP (control) or YFP-S100A11 were subjected to immunoprecipitation (IP) reactions using GFP Selector beads. Experiments were performed in the presence of Ca^2+^ to maintain the active Ca^2+^ bound conformation of S100A11 (Supplementary Figure S5). Screening for co-immunoprecipitated endogenous ANX across the samples demonstrated strong and specific interactions between S100A11 and ANXA1 (Supplementary Figure S5A,B; second graph from left) as well as ANXA2 (Supplementary Figure S5A,B; second graph from right). In contrast, ANXA6 did not co-immunoprecipitate with S100A11 (Supplementary Figure S5A,B; rightmost graph).

**FIGURE 3:**
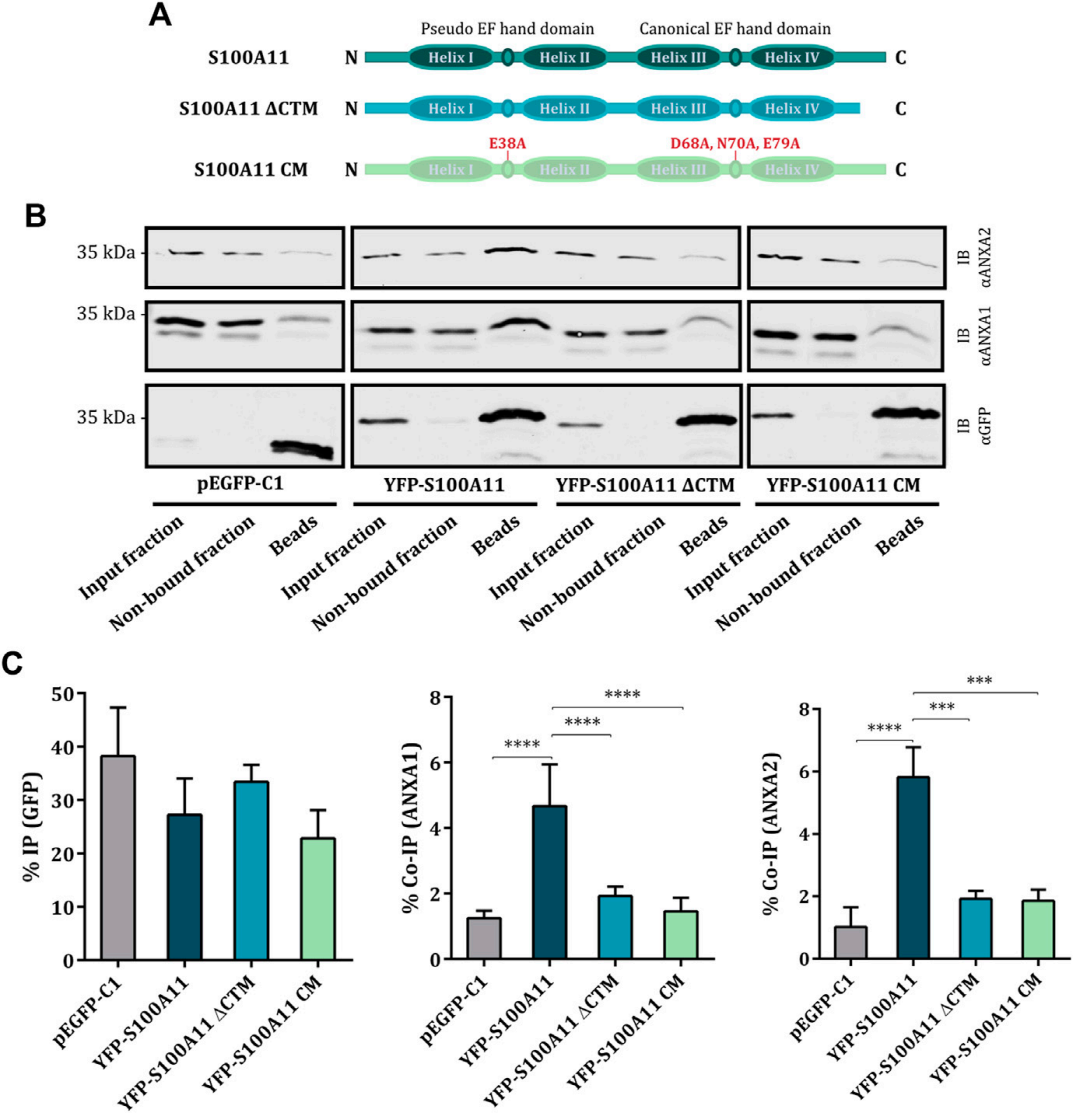
S100A11 interacts with ANXA1 and ANXA2 *via* its C-terminal extension at elevated Ca^2+^ conditions. **(A)** Domain structures of wild type S100A11, S100A11 ΔCTM and S100A11 CM. The scheme shows N-terminus on the left and C-terminus on the right. **(B)** Representative immunoblots of samples (input fraction, non-bound fraction and immunoprecipitates/beads) from S100A11-KO cells transfected with empty GFP vector, YFP-S100A11, YFP-S100A11 ΔCTM or YFP-S100A11 CM. Lysates of the respective cells were subjected to IP reactions in the presence of 2 mM Ca^2+^. Immunoblots were probed with anti-GFP antibodies to detect GFP alone (27 kDa) and YFP-S100A11, YFP-S100A11 ΔCTM or YFP-S100A11 CM (∼39 kDa), respectively, and with anti-ANXA1 (39 kDa) or anti-ANXA2 (38 kDa) antibodies to detect co-immunoprecipitated endogenous proteins. Representative blots from three to six independent experiments are shown. Note that ANXA1 and ANXA2 show some unspecific binding to the beads in control experiments carried with the empty GFP vector. This background binding is also observed in the YFP-S100A11 ΔCTM and YFP-S100A11 CM immunoprecipitates, but is significantly lower than the Co-IP in case of YFP-S100A11. **(C)** IP efficiencies for GFP, YFP-S100A11, YFP-S100A11 ΔCTM, and YFP-S100A11 CM, respectively (left), and Co-IP efficiencies for endogenous ANXA1 (middle) or ANXA2 (right) quantified in three to six independent blots. Statistical comparisons between groups were performed with ordinary one-way ANOVA (Tukey’s multiple comparison test). Data are mean ± SD.

S100 proteins, including S100A11, are proposed to associate with ANX via their C-terminal extension, a unique sequence following the F-helix of the second EF hand which is exposed in the Ca^2+^ bound conformation (Réty et al., 2000; Rintala-Dempsey et al., 2008; Hung et al., 2012). Based on these considerations, we generated two different S100A11 mutants to investigate the molecular details of the association of S100A11 with ANXA1 and ANXA2. A stop codon was inserted after amino acid D93 to obtain the S100A11 C-terminal deletion mutant (S100A11 ΔCTM) lacking the ANX binding sequence. To eliminate the Ca^2+^ binding capacity, Ca^2+^ associating amino acids E38 in the N-terminal pseudo EF hand and D68, N70 and E79 in the C-terminal canonical EF hand were mutated to A (domain structures depicted in Figure 3A), generating the S100A11 Ca^2+^ mutant (S100A11 CM) defective in Ca^2+^ binding. The characteristics of these mutants and their interactions with ANX were analyzed in S100A11-KO EA.hy926 cells. Co-IP analysis revealed significant and specific interactions between S100A11 WT and ANXA1 (Figures 3B,C; middle graph) as well as ANXA2 (Figures 3B,C; right graph), as described above for HUVEC (Supplementary Figure S5). However, significantly compromised interactions with ANXA1 and ANXA2 were observed for both, S100A11 ΔCTM and S100A11 CM (Figures 3B,C; middle and right graphs). Thus, S100A11 interacts with ANXA1 and ANXA2 but not ANXA6 in endothelial cells and this interaction requires intact Ca^2+^-binding sites as well as the presence of the C-terminal extension sequence in S100A11.

### Loss of S100A11 disrupts the wound site recruitment of ANXA2 but not ANXA1

**FIGURE 4:**
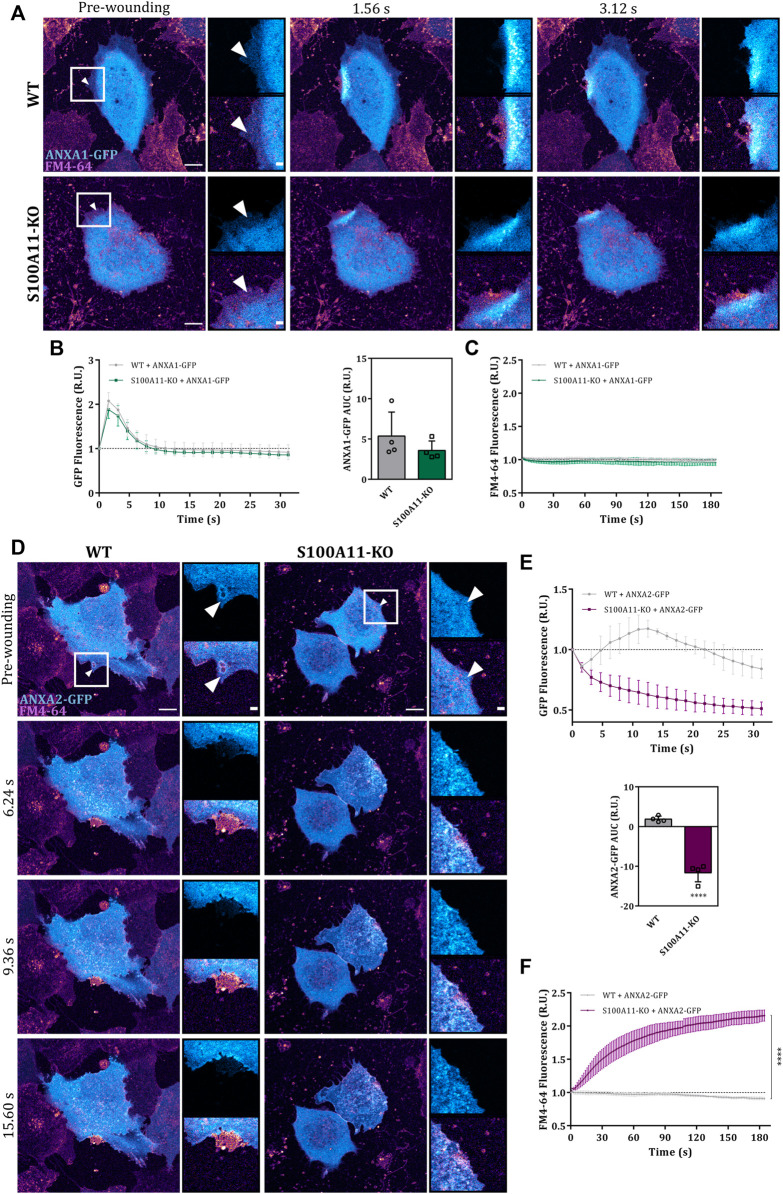
Absence of S100A11 affects the wound site recruitment of ANXA2 but not ANXA1. **(A)** Time-lapse images of ANXA1 recruitment to laser-induced plasma membrane wounds in WT or S100A11-KO cells, respectively (Supplementary Video S6). ANXA1-GFP expressing cells kept in Tyrode’s buffer supplemented with 2.5 mM Ca^2+^ and 5 μg/ml FM4-64 were injured at the lateral membrane edge (white triangles represent the wound sites). Wounding occurred at t = 0; pre-wounding and post-wounding time points are shown. Scale bars = 10 µm. Insets highlight the immediate vicinity of the wound. Scale bars = 2 μm. **(B)** Time course of ANXA1-GFP fluorescence in WT or S100A11-KO cells, normalized to fluorescence before injury. Mean fluorescent intensity was measured in the area next to the wound site (a circular ROI of 10 μm around the wound) for each acquisition time point. ANXA1-GFP AUCs for WT and S100A11-KO cells are also shown. Results were pooled from four independent experiments (*n* = 24). Statistical analysis was performed with two-tailed Student’s t test. **(C)** Time course of whole-cell FM4-64 fluorescence in WT or S100A11-KO cells ectopically expressing ANXA1-GFP, normalized to fluorescence before injury. Control experiments carried out in the absence of extracellular Ca^2+^ (EGTA containing medium) showed no resealing under the experimental condition used here, indicative of a still Ca^2+^-dependent repair process. Statistical comparison between FM4-64 AUCs was performed with two-tailed Student’s t test. **(D–F)** Same as in **(A–C)**, but WT or S100A11-KO cells were transfected with ANXA2-GFP (Supplementary Video S7). Data are mean ± SD.

As ANX play important roles in endothelial membrane wound repair (Koerdt and Gerke, 2017), we next assessed whether the compromised membrane resealing in S100A11-KO cells was associated with abnormal trafficking of its ANX interaction partners, ANXA1 or ANXA2, to the site of injury. Therefore, we examined the response of GFP tagged ANXA1 or ANXA2 to laser damage in WT and S100A11-KO cells. Immunoblot analysis showed that protein levels of ANXA1-GFP or ANXA2-GFP were similar in WT and S100A11-KO cells (Supplementary Figure S6A). In WT cells, ANXA1-GFP translocated rapidly to the wound site (within 2 s) and returned to the pre-wounding state within 5–10 s after injury (Figure 4A), whereas ANXA2-GFP exhibited a slower recruitment that started at around 3–5 s and returned to baseline within 15–20 s post-injury (Figure 4D). In both cases, punctate structures appeared in the wavefront similar to those observed for S100A11. In S100A11-KO cells, the recruitment kinetics of ANXA1-GFP were nearly identical (Figures 4A,B), indicating that S100A11 does not affect the recruitment of ANXA1 to membrane wounds in endothelial cells. Interestingly, as seen with the FM4-64 dye uptake assay, ectopic expression of ANXA1-GFP in S100A11-KO cells reduced the repair defect (Figures 4A,C). In contrast to ANXA1, wound-associated translocation of ANXA2-GFP was affected significantly by loss of S100A11 (Figures 4D,E). This is also seen in the AUC values for the ANXA2-GFP translocation time courses which were significantly smaller in S100A11-KO as compared to WT cells (Figure 4E). However, in contrast to ANXA1, ectopic expression of ANXA2-GFP in S100A11-KO cells had no effect on the repair defect (Figures 4D,F). To verify that the wound-directed recruitment and repair effects seen in S100A11-KO cells expressing ANXA1- or ANXA2-GFP were not due to altered expression of the endogenous proteins, we performed immunoblot analyses of WT and S100A11-KO cell lysates. Protein levels of endogenous ANXA1 and ANXA2 were nearly equivalent in WT and S100A11-KO cells (Supplementary Figure S6B,C) indicating that deletion of S100A11 is not compensated by an upregulation of ANXA1 or ANXA2 expression levels.

### The C-terminal extension of S100A11 is dispensable for its role in plasma membrane repair

**FIGURE 5:**
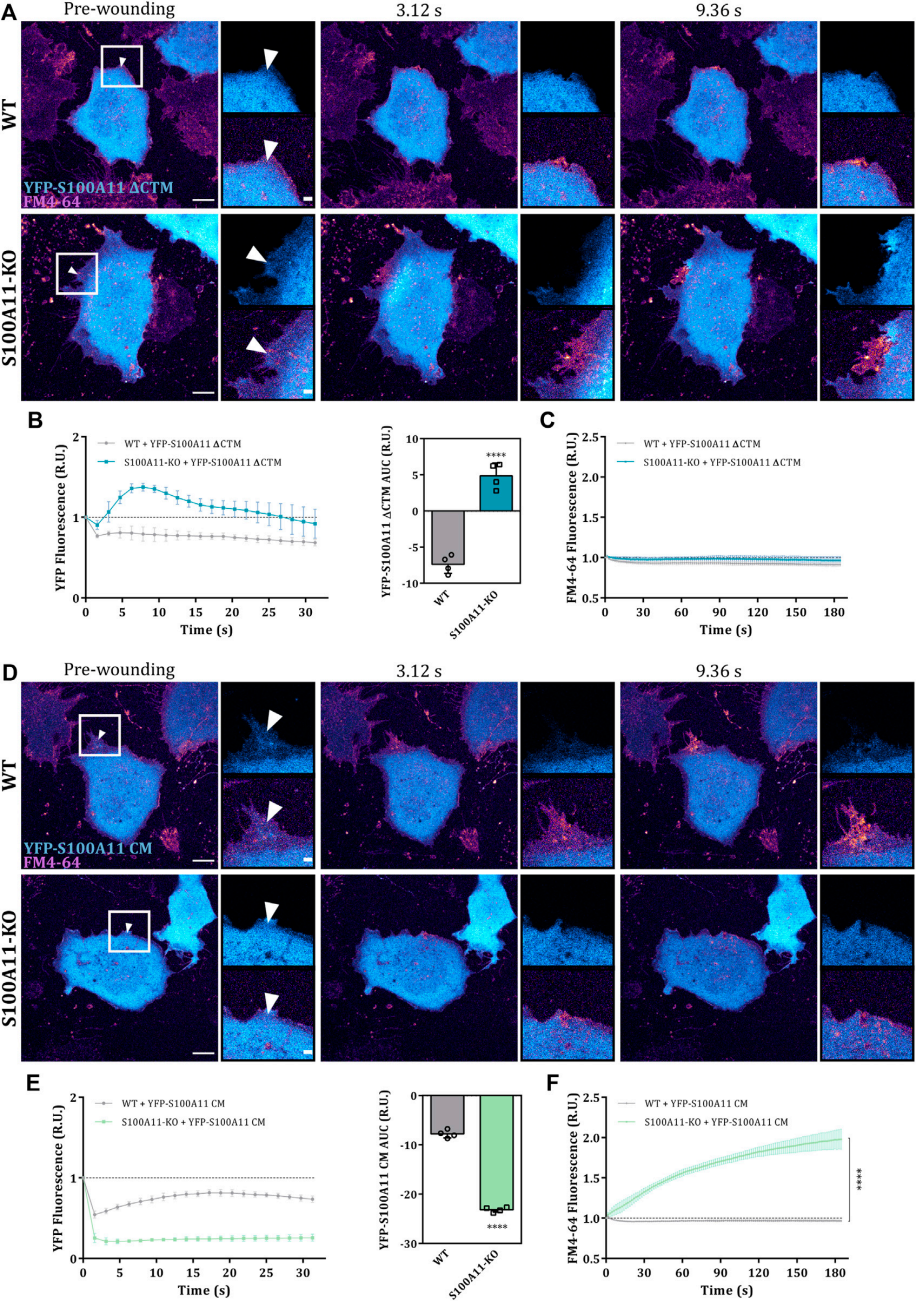
The C-terminal extension is dispensable for the function of S100A11 in PM repair. **(A)** Time-lapse images of S100A11 ΔCTM dynamics in response to laser-induced plasma membrane wounds in WT or S100A11-KO cells, respectively (Supplementary Video S8). YFP-S100A11 ΔCTM expressing cells kept in Tyrode’s buffer supplemented with 2.5 mM Ca^2+^ and 5 μg/ml FM4-64 were injured at the lateral membrane edge (white triangles represent the wound sites). Wounding occurred at t = 0; pre-wounding and post-wounding time points are shown. Scale bars = 10 µm. Insets highlight the immediate vicinity of the wound. Scale bars = 2 μm. **(B)** Time course of YFP-S100A11 ΔCTM fluorescence in WT or S100A11-KO cells, normalized to fluorescence before injury. Mean fluorescent intensity was measured in the area next to the wound site (a circular ROI of 10 μm around the wound) for each acquisition time point. YFP-S100A11 ΔCTM AUCs for WT and S100A11-KO cells are also shown. Results were pooled from four independent experiments (*n* = 24). Statistical analysis was performed with two-tailed Student’s t test. **(C)** Time course of whole-cell FM4-64 fluorescence in WT or S100A11-KO cells ectopically expressing YFP-S100A11 ΔCTM, normalized to fluorescence before injury. Control experiments carried out in the absence of extracellular Ca^2+^ (EGTA containing medium) showed no resealing under the experimental condition used here, indicative of a still Ca^2+^-dependent repair process. Statistical comparison between FM4-64 AUCs was performed with two-tailed Student’s t test. **(D–F)** Same as in **(A–C)**, but WT or S100A11-KO cells were transfected with YFP-S100A11 CM (Supplementary Video S9). Data are mean ± SD.

Given that the C-terminal extension (residues 93–105) and the Ca^2+^-binding sites in S100A11 are crucial for interaction with its ANX partners, we tested whether the PM wound repair defect in S100A11-KO cells can be rescued by expression of the S100A11 ΔCTM or S100A11 CM mutants. First, we verified by immunoblot analysis that the expression levels of YFP-S100A11, YFP-S100A11 ΔCTM and YFP-S100A11 CM were nearly identical in WT or S100A11-KO cells and comparable amongst each other (Supplementary Figure S7A). Similar to wild type S100A11, the mutants also localized to the cytosol of endothelial cells (Figures 5A,D). However, in contrast to the wild type protein, S100A11 ΔCTM and S100A11 CM failed to display significant wound-associated dynamics in WT cells (Figures 5A,D). Quantification of S100A11 ΔCTM and S100A11 CM kinetics only revealed a reduction in fluorescence intensity following injury, which could be attributed to bleaching or loss of cytoplasmic YFP-tagged molecules (Figures 5B,E). It should be noted that the ectopic expression of neither S100A11 ΔCTM nor S100A11 CM affected PM wound repair efficiency of WT cells (Figures 5A,C,D,F), which indicates that expression of the mutants does not result in a dominant-negative phenotype, at least not at the expression levels achieved in this study.

In S100A11-KO cells, contrary to our expectations, S100A11 ΔCTM translocated to the wound site although to a somewhat lesser extent as compared to the wild type protein (Figures 5A,B). AUC values for the S100A11 ΔCTM time courses showed significant recruitment in S100A11-KO cells but were smaller than those of wild type S100A11 (Supplementary Figure S7B; left graph). Most likely, this difference to WT cells is due to the fact that PM binding sites for S100A11 ΔCTM are occupied by wild type S100A11 in the WT cells. Importantly, expression of S100A11 ΔCTM was sufficient to rescue the repair defect in S100A11-KO cells (Figures 5A,C and Supplementary Figure S7B; right graph). Thus, PM wound repair proceeds independently of interactions between S100A11 and ANX in S100A11-KO cells expressing fluorescently tagged S100A11 ΔCTM. The other mutant S100A11 CM remained insensitive to wounding-induced Ca^2+^ influx in S100A11-KO cells (Figures 5D,E) and its AUC values were significantly smaller than those of wild type S100A11 (Supplementary Figure S7B). This shows that the wound site translocation of S100A11 requires Ca^2+^ binding to the EF hands of the protein. Moreover, and in contrast to S100A11 ΔCTM, S100A11 CM was not able to rescue the membrane resealing defect in S100A11-KO cells as revealed by the unaltered high FM4-64 dye uptake following laser ablation (Figures 5D,F). In addition, a significant reduction of YFP-S100A11 CM fluorescence was observed in S100A11-KO as compared to WT cells, presumably as a result of cytoplasmic leakage through the non-repaired membrane wounds (Figures 5D,E). We conclude that the Ca^2+^-binding sites but not the ANX binding C-terminal extension of S100A11 are indispensable for its wound-associated recruitment and positive regulatory role in endothelial membrane wound repair.

### Extended synaptotagmin (E-Syt1), a novel interaction partner of S100A11 in endothelial cells

**FIGURE 6:**
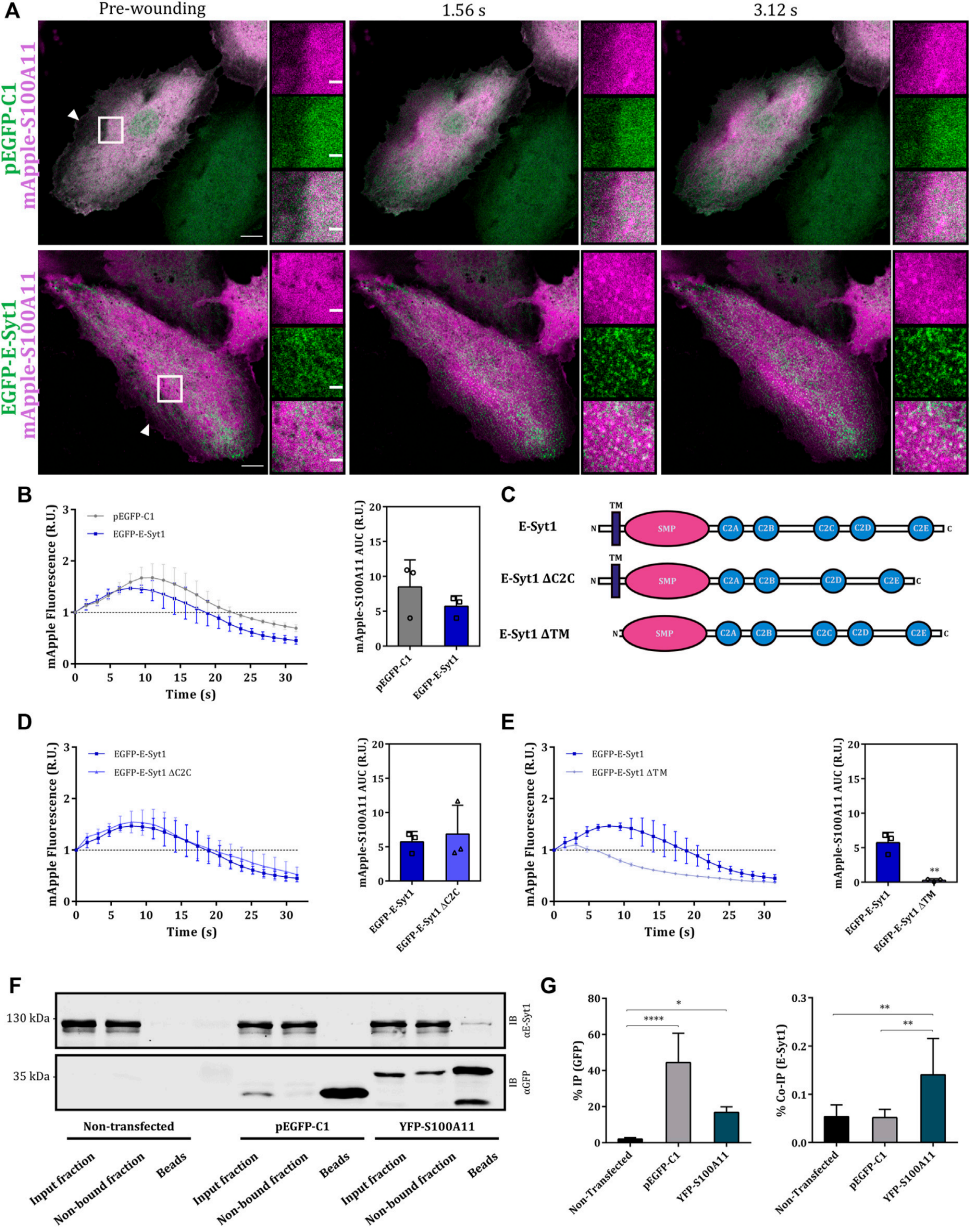
S100A11 associates with E-Syt1 in endothelial cells. **(A)** Representative time-lapse images of injured HUVEC expressing GFP plus mApple-S100A11 (upper panel) or EGFP-E-Syt1 plus mApple-S100A11 (lower panel) (Supplementary Video S11). Transfected cells kept in Tyrode’s buffer supplemented with 2.5 mM Ca^2+^ were injured at the lateral membrane edge (white triangles represent the wound sites). Wounding occurred at t = 0; pre-wounding and post-wounding time points are shown. Scale bars = 10 µm. Insets highlight the immediate vicinity of the wound. Scale bars = 2 μm. **(B)** Time course of mApple-S100A11 fluorescence in injured HUVEC expressing GFP or EGFP-E-Syt1, normalized to fluorescence before injury. mApple-S100A11 AUCs for empty GFP vector or EGFP-E-Syt1 transfected HUVEC are also shown. Results were pooled from three independent experiments (*n* = 18). Statistical analysis was performed with two-tailed Student’s t test. **(C)** Domain structures of wild type E-Syt1 as well as E-Syt1 ΔC2C and E-Syt1 ΔTM mutants. The scheme shows N-terminus on the left and C-terminus on the right. **(D,E)** Time course of mApple-S100A11 fluorescence in EGFP-E-Syt1 ΔC2C (D) or EGFP-E-Syt1 ΔTM (E) expressing HUVEC, normalized to fluorescence before injury. mApple-S100A11 AUCs for EGFP-E-Syt1 ΔC2C or EGFP-E-Syt1 ΔTM transfected HUVEC are also shown. Results were pooled from three independent experiments (*n* = 18). Statistical analyses were performed with two-tailed Student’s t test. **(F)** Representative immunoblots of samples (input fraction, non-bound fraction and immunoprecipitates/beads) from non-transfected HUVEC or HUVEC transfected with GFP or YFP-S100A11 plasmids, which were subjected to IP reactions in the presence of 2 mM Ca^2+^. Immunoblots were probed with anti-GFP antibodies to detect GFP alone (27 kDa) and YFP-S100A11 (39 kDa), and with anti E-Syt1 (123 kDa) antibodies to detect co-immunoprecipitated endogenous protein. Representative blots from seven independent experiments are shown. **(G)** IP efficiencies for empty GFP vector, YFP-S100A11 (left) and Co-IP efficiencies for endogenous E-Syt1 (right) quantified in seven independent blots. Statistical comparisons between groups were performed with ordinary one-way ANOVA (Tukey’s multiple comparison test). Data are mean ± SD.

As S100A11 trafficking and function in the wounding response can occur independent of binding to ANX, we hypothesized that (an)other, most likely Ca^2+^-regulated membrane binding protein may interact with S100A11 and coordinate its membrane translocation in the course of PM wounding and resealing. One class of such candidate proteins are extended synaptotagmins (E-Syts), tether proteins that regulate endoplasmic reticulum-plasma membrane (ER-PM) contact sites in response to changes in cytosolic Ca^2+^ (for review see Saheki and De Camilli, 2017). E-Syts are characterized by a synaptotagmin-like mitochondrial-lipid-binding protein domain (SMP) flanked by an N-terminal ER-membrane anchor/transmembrane (TM) and cytosolic C2 domains that sense Ca^2+^ fluctuations (Min et al., 2007). As E-Syt1 is specifically recruited to ER-PM contact sites in response to Ca^2+^ rise (Giordano et al., 2013), we first analyzed its behaviour in response to membrane wounding by recording the dynamics of EGFP tagged E-Syt1 in injured endothelial cells. In HUVEC, E-Syt1 showed a uniform ER distribution and following membrane injury, translocated rapidly to punctate structures, most likely resembling junctions between ER and PM (Giordano et al., 2013; Supplementary Figure S8A; insets). E-Syt1 punctae were more pronounced in the proximity of the wound and punctae formation required the wounding-induced influx of Ca^2+^ as it was not observed when HUVEC were wounded in the absence of extracellular Ca^2+^ (Supplementary Figure S8B). We next compared the kinetics of wound-associated S100A11 and E-Syt1 dynamics in cells ectopically expressing mApple tagged S100A11 and EGFP-E-Syt1 (Figures 6A,B). Interestingly, we observed an immediate but transient colocalization of S100A11 with the Ca^2+^-induced E-Syt1 punctae that most likely represent ER-PM contact sites (Figures 6A).

To further characterize the wounding-induced E-Syt1 dynamics and its association with S100A11 translocation, we generated E-Syt1 deletion mutants lacking the TM (amino acids 1–91) or the pivotal C2 domain, C2C (amino acids 627–751) (domain structures are depicted in Figure 6C) as E-Syt1 requires both, the TM and the C2C domains for establishing ER-PM contacts (Giordano et al., 2013; Yu et al., 2016). In resting HUVEC, E-Syt1 ΔC2C, which was expressed at low levels, localized to the ER (Supplementary Figure S8C), whereas EGFP-E-Syt1 ΔTM showed a general cytosolic distribution (Supplementary Figure S8D). In contrast to what was observed for full-length E-Syt1, PM wounding did not induce the formation of punctae containing E-Syt1 ΔC2C, likely explained by its reduced Ca^2+^ sensitivity (Supplementary Figure S8C). Interestingly, upon wounding, E-Syt1 ΔTM displayed bright punctate structures immediately followed by a subtle wave-like recruitment towards the wound site within 30–40 s post-wounding (Supplementary Figure S8D). This behaviour is probably mediated by the C2 domains in E-Syt1 ΔTM which are capable of Ca^2+^-dependent membrane binding. Next, we analyzed the kinetics of wound site recruitment of S100A11 in cells expressing the E-Syt1 mutants. Expression of E-Syt1 ΔC2C did not have a significant effect as S100A11 recruitment kinetics were comparable to those in cells expressing wild type E-Syt1 (Figure 6D and Supplementary Figure S9; upper panel). On the other hand, S100A11 recruitment was significantly attenuated upon expression of EGFP-E-Syt1 ΔTM (Figure 6E and Supplementary Figure S9; lower panel). This was corroborated by the AUC values for S100A11 translocation time courses: mApple-S100A11 AUCs in HUVEC co-expressing E-Syt1 ΔC2C were nearly identical to those in cells expressing wild type E-Syt1, whereas the AUCs in cells co-expressing E-Syt1 ΔTM were significantly smaller (Figures 6D,E). These results show that membrane wound-directed recruitment of S100A11 appears to be associated with ER-PM contact site-localized E-Syt1 in endothelial cells.

**FIGURE 7:**
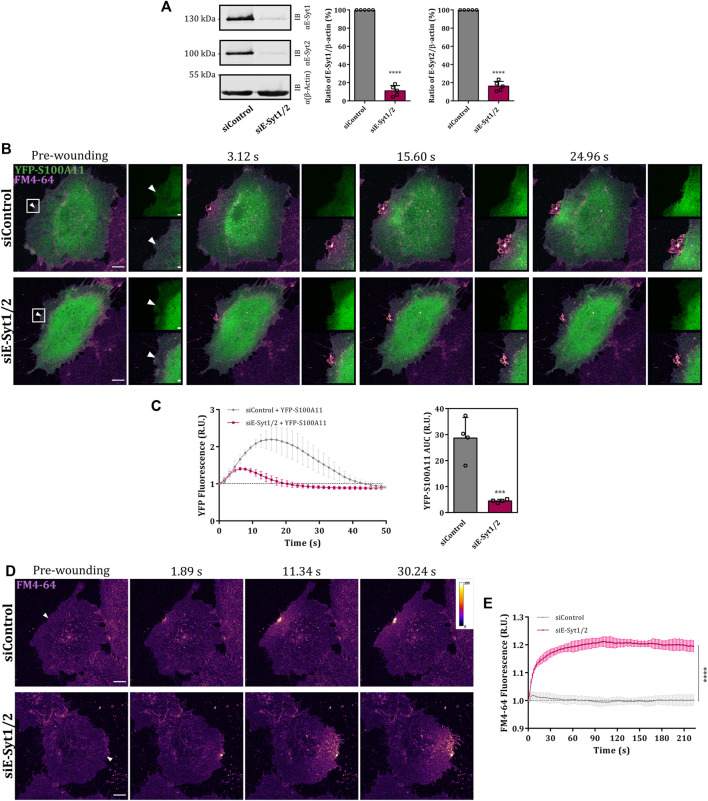
E-Syt1 regulates translocation of S100A11 to plasma membrane wounds. **(A)** Immunoblot analysis of endogenous E-Syt1 (124 kDa) and E-Syt2 (102 kDa) expression in HUVEC transfected with control or E-Syt1 and E-Syt2-specific siRNAs (siControl or siE-Syt1/2, respectively). β-actin (42 kDa) is shown as the loading control. Representative blots from five independent experiments are shown. Efficiencies of E-Syt1 and E-Syt2 knockdown quantified in five independent blots are also shown. Statistical comparisons between non-targeted and targeted knockdowns were performed with two-tailed Student’s t test. **(B)** Time-lapse images of S100A11 recruitment to laser-induced plasma membrane wounds in siControl or siE-Syt1/2 treated HUVEC (Supplementary Video S12). Cells kept in Tyrode’s buffer supplemented with 2.5 mM Ca^2+^ and 5 μg/ml FM4-64 were injured at the lateral membrane edge (white triangles represent the wound sites). Wounding occurred at t = 0; pre-wounding and post-wounding time points are shown. Scale bars = 10 µm. Insets highlight the immediate vicinity around the wound. Scale bars = 2 μm. **(C)** Time course of YFP-S100A11 fluorescence in siControl or siE-Syt1/2 treated HUVEC, normalized to fluorescence before injury. Mean fluorescent intensity was measured in the area next to the wound site (a circular ROI of 15 μm around the wound) for each acquisition time point. YFP-S100A11 AUCs for siControl or siE-Syt1/2 treated HUVEC are also shown. Results were pooled from four independent experiments (*n* = 40). Statistical analysis was performed with two-tailed Student’s t test. **(D)** Representative images of FM4-64 infiltration following membrane damage (white triangles represent the wound sites) in HUVEC transfected with control or E-Syt1 and E-Syt2-specific siRNAs (Supplementary Video S13, S14). Calibration bar of fluorescence intensity is provided on the upper right panel. Scale bars = 10 µm. **(E)** Time course of whole-cell FM4-64 fluorescence in HUVEC transfected with control or E-Syt1 and E-Syt2-specific siRNAs, normalized to fluorescence before injury. Data were compared for AUC values of FM4-64 fluorescence. Control experiments carried out in the absence of extracellular Ca^2+^ (EGTA containing medium) showed no resealing under the experimental condition used here, indicative of a still Ca^2+^-dependent repair process. Results were pooled from three independent experiments (*n* = 36). Statistical analysis was performed with two-tailed Student’s t test. Data are mean ± SD.

In view of this transient wounding-induced colocalization, we next analyzed whether S100A11 and E-Syt1 can interact with one another in the presence of Ca^2+^. Co-IP experiments employing YFP-S100A11 expressed in HUVEC revealed a low but significant and specific interaction with endogenous E-Syt1 (Figures 6F,G; right graph). Given this interaction, we assessed whether E-Syt1 could be the factor mediating the wound site translocation of S100A11 and its function in PM repair. Therefore, we depleted HUVEC of E-Syt1 and E-Syt2 (to exclude compensatory effects by this related family member) by siRNA (Figure 7A) and analyzed wound site recruitment of YFP-S100A11. As shown in Figures 7B,C, wounding-induced translocation of YFP-S100A11 is markedly reduced in E-Syt1/2 downregulated cells. Next, PM resealing was assessed in cells depleted of E-Syt1/2 by employing the FM4-64 dye uptake assay. While cells treated with control siRNA showed proper resealing, i.e. restriction of dye uptake within the first seconds post wounding, cells lacking E-Syt1/2 exhibited continuous FM4-64 uptake typically observed in resealing defective conditions (Figures 7D,E). Thus, proper PM wound resealing in endothelial cells requires the presence of E-Syt1 which most likely functions in conjunction with S100A11 that is recruited to the wound site in an E-Syt1-dependent manner.

## Discussion

The resealing of plasma membrane wounds is an essential process enabling cell survival after injury. Ca^2+^ entering through the wound serves as the prime initiator of the cell-autonomous resealing response, and many Ca^2+^-sensing proteins have been implicated as part of the PM repair machinery. They include several annexins, in particular ANXA1, A2, A5, A6 and A7, as well as their interaction partners (Boye and Nylandsted, 2016; Koerdt et al., 2019). One of these interaction partners, S100A11, is of prime interest as it can also bind Ca^2+^ through its EF hands and has been shown to interact with two annexins, ANXA1 and A2 (Rintala-Dempsey et al., 2008; Jaiswal et al., 2014). Confirming earlier studies employing siRNA-mediated knockdown (Jaiswal et al., 2014; Ashraf and Gerke, 2021), we show by a complete knockout approach that S100A11 is a critical player of PM wound repair in endothelial cells. This activity as well as the rapid translocation of S100A11 to the wound site requires intact EF hand-type Ca^2+^-binding sites, suggesting that recruitment of S100A11 to the site of injury is an active process stimulated by wound-induced increase in cytosolic Ca^2+^ levels. As S100A11 is not a membrane binding protein per se, wound site recruitment likely involves a (Ca^2+^-dependent) interaction with a membrane targeting protein, followed by a lateral movement towards the wound. The latter could involve continuous dissociation from and Ca^2+^-dependent reassociation to the PM which manifest themselves in the form of dynamic waves. A similar wave-like recruitment to the site of injury is observed for ANX in endothelial cells (Koerdt and Gerke, 2017; Ashraf and Gerke, 2021), and thus ANX, in particular the known interaction partners ANXA1 and A2, probably serve as candidates for mediating a wounding-induced membrane translocation of S100A11. While ANXA1 has been described as the canonical binding partner of S100A11 (Réty et al., 2000; Rintala-Dempsey et al., 2008), ANXA2 (Rintala-Dempsey et al., 2006; Streicher et al., 2009) and ANXA6 (Chang et al., 2007; Rintala-Dempsey et al., 2008) have also been reported to interact with S100A11. We show here that in endothelial cells, Ca^2+^-dependent interactions occur between S100A11 and ANXA1 and A2, excluding ANXA6 from being involved in the S100A11 translocation. Interactions between S100A11 and ANXA1 and A2 result in heterotetrameric complexes that could link two membrane surfaces via the membrane binding ANX moieties suggesting that such membrane linkage, e.g. between intracellular vesicles and the PM and/or between torn PM edges could be involved in the resealing process (Boye and Nylandsted, 2016; Koerdt et al., 2019). ANXA1 and A2 can also bind to F-actin (Gerke et al., 2005) and thus, heterotetrameric complexes with S100A11 could also provide a link to the cortical actin cytoskeleton that needs to be modulated in the course of membrane repair.

ANXA1 and A2 have been shown to function in membrane resealing in HeLa cells and HUVEC, respectively (McNeil et al., 2006; Koerdt and Gerke, 2017). As our data show that wound site translocation of ANXA2 but not ANXA1 is compromised in S100A11-KO cells, we propose that the relevant annexin binding partner of S100A11 in the course of membrane repair in endothelial cells is ANXA2. This is consistent with a previous report implicating S100A11-ANXA2 complexes in PM wound repair and survival of metastatic cancer cells (Jaiswal et al., 2014). However, additional S100A11 binding partner(s) are likely involved as a S100A11 mutant, which lacks the ANX binding C-terminal extension (the S100A11 ΔCTM mutant) and does not bind ANX in endothelial cells, is still recruited to the wound site and importantly, can rescue the membrane repair defect in S100A11-KO cells. In search for such novel binding partner, we here identify E-Syt1, a Ca^2+^-regulated ER-PM tether. Cytosolic Ca^2+^ elevation triggers PM binding of ER-resident E-Syt1 and the formation of E-Syt1-mediated ER-PM contacts (Saheki and De Camilli, 2017). These regulated contacts most likely also form in response to PM wounding, which results in a substantial increase in cytosolic Ca^2+^ and initiates the formation of E-Syt1 punctae probably representing ER-PM contacts. Interestingly, the S100A11 translocation to endothelial membrane wound sites is also characterized by the appearance of static but short-lived (few seconds) punctate structures. These colocalize with the E-Syt1 positive punctae and thus could represent a Ca^2+^-triggered transient association of S100A11 with E-Syt1 at ER-PM contacts. In line with an involvement of E-Syt1 in mediating a PM translocation of S100A11 in response to wounding, depletion of E-Syt1 and E-Syt2 abrogates this translocation. In addition, we identified a compromised translocation of S100A11 upon ectopic expression of an E-Syt1 mutant lacking the ER membrane anchor, presumably because this mutant could act in a dominant-negative manner competing with endogenous E-Syt1. The data implying E-Syt1 as a PM recruitment factor of S100A11 in endothelial cells are supported by our observation that endogenous E-Syt1 is co-immunoprecipitated with S100A11. Together, these data suggest that E-Syt1 is an additional binding partner of S100A11 that regulates its translocation to the PM in response to wounding.

We not only identify E-Syt1 as a novel binding partner of S100A11, we also show that E-Syts are required to support PM resealing in endothelial cells as depletion of E-Syt1 and E-Syt2 significantly interferes with the repair of laser ablation-induced PM wounds. Mechanistically, it is not known how E-Syts support PM resealing. E-Syts could function independently of S100A11 by providing ER-PM tethers, which could for example aid the exchange of PM lipids required for efficient wound repair. Alternatively, or additionally, the role of E-Syt1 could be linked to S100A11 by mediating its PM translocation in response to wounding. Such translocation to ER-PM contacts could facilitate S100A11-ANXA2 complex formation at the PM which in turn is required for movement to the actual wound site and a function of this complex at the wound (see above). The latter is supported by the finding that wound site recruitment of ANXA2 also involves the formation of punctate structures that could represent the E-Syt1 positive ER-PM contact sites. Tripartite complexes comprising ANXA2, S100A11 and E-Syt1 could form as the binding sites of E-Syt1 on S100A11 likely does not involve the C-terminal extension that constitutes the ANXA2 binding site. Once recruited to ER-PM contacts, ANXA2-S100A11 complexes might even further stabilize the ER-PM tethering in a manner similar to what has been reported for ANXA1-S100A11 complexes at ER-endosome contact sites of HeLa cells (Eden et al., 2016). However, it has to be noted that the association of S100A11 and ANXA2 with the E-Syt1 punctae is transient and occurs before a longer-lived concentration at the actual wound site. Further investigations on the dynamics of ER-PM contact sites in injured cells in the absence of ANXA2 and/or S100A11 have to clarify this point.

On the basis of our findings, we propose a multi-step model for the dynamics and role of S100A11 and its interaction partners in PM wound repair (Supplementary Figure S10). In resting cells, ANX and S100A11 homodimers are predominantly cytosolic, whereas E-Syt1 is localized to the ER membrane. Cell injury leads to PM wound-associated Ca^2+^ influx, which triggers the formation of ER-PM contact sites rich in Ca^2+^-bound E-Syt1. The increase in cytosolic Ca^2+^ also activates S100A11 dimers via Ca^2+^ binding to the canonical C-terminal EF hands, thereby establishing a conformation capable of interacting with E-Syt1. This Ca^2+^ conformation can also interact with annexins A1 and A2 and our data suggest that following E-Syt1 mediated PM translocation, Ca^2+^-S100A11 interacts with ANXA2 which is recruited to the site of PM injury by Ca^2+^-dependent binding to acidic phospholipids [such as PS and PI(4,5)P_2_]. Here, the E-Syt1-dependent (and Ca^2+^-induced) recruitment of S100A11 to the PM (induced by Ca^2+^ binding to S100A11) precedes the wound site recruitment ANXA2 (induced by Ca^2+^ binding to ANXA2). Following the initial recruitment and thereby an increased local concentration at the PM, heterotetrameric S100A11-ANXA2 complexes form, possibly due to a somewhat higher S100A11 affinity of ANXA2 as compared to E-Syt1. These complexes are found enriched at and close to the wound site where the local ANXA2 concentrations are highest. At the actual wound site, the S100A11-ANXA2 heterotetramers, possibly in conjunction with other annexins and ANX-S100 complexes, could function in membrane repair by preferentially associating with the wound edges of the PM and supporting the formation of membrane bridges and/or a bending of the free membrane edges that eventually supports the actual resealing [as proposed, e.g., by (Bendix et al., 2020)]. Such functions would also be strictly Ca^2+^-dependent as Ca^2+^-free S100A11 will dissociate from ANXA2. Overall, our findings suggest that the relevance of S100A11 in PM wound repair extends beyond its putative function as an interaction partner of ANXA1 and A2.

## Materials and methods

### Plasmids and siRNAs

The following plasmids used in this study have been described previously: YFP-S100A11 (Seemann et al., 1997), ANXA1-GFP and ANXA2-GFP (Rescher et al., 2000). EGFP-E-Syt1 (Giordano et al., 2013) was purchased from Addgene (#66830), kindly deposited by Pietro De Camilli. mApple-S100A11 was generated by cloning the S100A11 cDNA sequence flanked by EcoRI and AgeI restriction sites (750 bp long insert) into the mApple-C1 vector (Addgene; #66830). Restriction enzymes and T4 ligase were purchased from New England BioLabs and used according to the manufacturer’s specifications.

S100A11 and E-Syt1 mutants were generated from YFP-S100A11 and EGFP-E-Syt1, respectively, by site-directed mutagenesis using the Q5 Site-Directed Mutagenesis Kit (New England BioLabs). To generate specific and targeted changes, following mutagenesis primers were used: S100A11 ΔCTM (# 1: 5′-CTT CCT CAA GGC TGT CCC TTC CCA GAA GCG GA-3′, # 2: 3′-GAT TAA TGG CAA GCC ATA GCT AGG CCA CCA ATC-5′), E-Syt1 ΔTM (# 1: 5′-CGC CGG GTC CGC GAC GAG-3′, # 2: 3′-GTC GAC TGC AGA ATT CGA AGC TTG AGC TC-5′) and E-Syt1 ΔC2C (# 1: 5′-ACC CTG GAG GAT GTC CCA-3′, # 2: 3′-GCT GCC TCT CTG GGG ATT C-5′). S100A11-CM was generated in two consecutive steps using the following primers: S100A11_E38A (# 1: 5′-CCT AAG CTT CAT GAA TAC AGA ACT AGC TGC CTT C-3′, # 2: 3′-AAC GCT GTC TTG GAG AGA GTG TAG TTA TAA CCA TCC-5′), S100A11_D68A, N70A, E79A (# 1: 5′-AGC TAG ATT TCT CAG CAT TTC TTA ATC TGA TTG GTG GCC TAG C-3′, # 2: 3′-GAC CAT CAC TGG CGG TGG CCA GTT TCT TCA TCA TGC GGT CAA GG-5′). All mutant constructs were verified by DNA sequencing.

The following siGENOME SMARTpool siRNAs (a mixture of 4 siRNAs provided as a single reagent) from Horizon Discovery were used in this study: human S100A11 (M-012138-00; # 1: 5′-CAA CAG UGA UGG UCA GCU A dTdT-3′, # 2: 5′-CUA CAG AGA CUG AGC GGU G dTdT-3′, # 3: 5′-UCG AGU CCC UGA UUG CUG U dTdT-3′, # 4: 5′-CUG GAA AGG AUG GUU AUA A dTdT-3′), human E-Syt1 (L-010652-00; # 1: 5′-GUA CUU GGA UUC AUC AGA A dTdT-3′, # 2: 5′-GUA CUA CAG UGA AGA ACG A dTdT-3′, # 3: 5′-CCA AGA CUA UUU CGC AAA C dTdT-3′, # 4: 5′-GCC CUG CUA UCC AUC UAU A dTdT-3′) and human E-Syt2 (L-025231-01; # 1: 5′-GGA CAG GAC UGA CGA AUC U dTdT-3′, # 2: 5′-CAA CUA AUU UCA CGU GAC A dTdT-3′, # 3: 5′-GGU AUG ACC UCA CGG AAG A dTdT-3′, # 4: 5′-AAU AUA UUC UGC ACG GUA A dTdT-3′). Control experiments employed AllStars Negative Control siRNA from Qiagen (1027281).

### Cell cultivation

HUVEC were obtained from PromoCell as cryo-conserved pools (C-12203) and cultivated on pre-coated Corning CellBIND dishes in 1:1 mixed medium comprising Endothelial Cell Growth Medium (ECGM2, PromoCell) supplemented with 10% FBS, 20 μg/ml gentamicin and 15 μg/ml amphotericin B and Medium 199 (M199, Sigma-Aldrich) supplemented with 10% FBS, 20 μg/ml gentamicin, 15 μg/ml amphotericin B. HUVEC in passage 3-5 were utilized for experiments. EA.hy926 wild type (WT) cells were kindly provided by Roland Weldich Söldner (University of Muenster) and cultured in Dulbecco’s Modified Eagles Medium (DMEM, Sigma-Aldrich) containing 10% fetal bovine serum (FBS), 1% L-Glutamine, 1% Penicillin-Streptomycin and 1% non-essential amino acids (NEAA). HUVEC and EA.hy926 cells were maintained at 37°C and 5% CO_2_ atmosphere.

### Generation of CRISPR/Cas S100A11 knockout cell lines

CRISPR/Cas knockout of the S100A11 gene (S100A11-KO) was generated in EA.hy926 cells. Therefore, a ready-to-use transfection-based S100A11 gene specific gRNA-Cas9 expression plasmid vector (S100A11 CRISPR gRNA 4_PX459 V2.0) from GenScript was utilized (Supplementary Figure S1A). The gRNA sequence (5′-GCT​GTC​TTC​CAG​AAG​TAT​GC-3′) was designed (Sanjana et al., 2014) to uniquely target the human S100A11 gene and plasmid vector resistance against puromycin was used to identify cells with gRNA and Cas9 expression. EA.hy926 cells seeded on multiple wells in a 24-well plate were transfected (using Lipofectamine 2000 as described below) with 0.8 µg of S100A11/Cas9-guide plasmid. 48 h post-transfection, cells were washed with PBS^+/+^ to remove dead cells and provided with fresh growth media for 6 h. Transfected cells were maintained in selection media containing 0.5 μg/ml puromycin (titrated concentration) for 5 days with stringent washing using PBS−/− every 24 h. Thereafter, for single-cell cloning, cells were pooled and serially diluted into a 96-well plate containing growth media. Clones were incubated at 37°C and 5% CO_2_ until each attained confluency. Around 40 clones were selected randomly and split or reseeded for continuous culture (96-well → 24-well → 6-well → 100 mm culture dishes). To evaluate the KO, each clone was seeded into a 150 mm culture dish, cell lysates were harvested and endogenous S100A11 expression was analyzed by immunoblotting. Efficiency of KO was calculated as a percentage of the ratio of the S100A11 signal intensity against the intensity of the loading control (β-actin). For all experiments using S100A11-KO cells, WT EA.hy926 cells were included as the negative control. Analyses were carried out with two independent clonal populations to rule out clonal artefacts. Results were basically identical and for clarity, only those for one clonal population are shown.

### Transient transfection

Distinct transfection methods were optimized for EA.hy926 cells and HUVEC in this work. EA.hy926 cells were subjected to plasmid DNA transfections using Lipofectamine 2000 (Invitrogen) according to the manufacturer’s instructions. Confluent adherent cells were incubated with appropriate amount of Opti-MEM-diluted DNA-lipofectamine complex (corresponding to the size of the cell culture dish) for 24 h until further processing. Transfections of HUVEC were performed using the Amaxa nucleofection system (Lonza) according to the manufacturer’s instructions. HUVEC from a confluent 60 mm culture dish were transfected with 1–4 μg plasmid DNA in self-made nucleofection buffer (4 mM KCl, 10 mM MgCl_2_, 10 mM sodium succinate, 100 mM NaH_2_PO_4_ pH 7.4). 18–24 h post transfection, HUVEC were used for different functional assays as described below. For siRNA mediated knockdown, HUVEC were transfected with the respective siRNA (500 pmol per 60 mm culture dish), cultivated for 48 h and then re-transfected with the same amount of siRNA. To ectopically express S100A11 in the knockdown background, the second round of siRNA transfection also included YFP-S100A11 (2 μg). 24 h post-transfection, knockdown efficiency was assessed by immunoblotting and represented as a percentage of the ratio of respective protein signal intensity against the loading control (β-actin).

### Antibodies

Primary antibodies and their dilutions utilized in this study: rabbit anti-S100A11 (1:1500, ProteinTech), rabbit anti-ANXA1 (1:2500, Invitrogen), mouse anti-ANXA2 (1:1000, Thiel et al., 1992), mouse anti-ANXA6 (1:1000, BD Transduction Lab), mouse anti-E-Syt1 (1:1000, ProteinTech), rabbit anti-E-Syt2 (1:1000, ProteinTech), rabbit anti-GFP (1:1000, Invitrogen), Living Colors mouse anti-GFP (1:1000, Takara Bio) and mouse anti-β-Actin (1:2000, Sigma-Aldrich).

### Immunoblotting

For production of cell lysates, cells harvested with trypsin/EDTA were washed in ice-cold PBS. Cell pellets from a 60 mm culture dish were resuspended in 40 μL lysis buffer containing 20 mM HEPES pH 7.4, 150 mM NaCl, 0.5% Triton X-100, 1.5 mM PMSF and Complete Protease Inhibitor Cocktail (Roche) and lysed for 1 min by sonication using a vial tweeter (0.5 cycle, 100% amplitude). The sonicated mixtures were incubated on ice for 15 min and centrifuged at 1250 x g for 10 min at 4°C. Protein concentrations of collected supernatants were determined using the Pierce 660 nm protein assay (Thermo Fisher Scientific). The protein samples were boiled at 95°C for 10 min in one-fifth volumes of 5x SDS sample buffer.

Lysate samples were subjected to SDS-PAGE in 10% or 15% gels for 45 min at 70–85 V and subsequently at 90–120 V. The separated proteins were transferred onto 0.2 µm nitrocellulose membranes in a wet tank system at 115 V for 1 h at 4°C using Tris-Glycine transfer buffer (25 mM Tris, 200 mM glycine, 20% (v/v) methanol). Membranes were blocked in TBS-T (20 mM Tris-HCl pH 7.5, 150 mM NaCl, 0.1% (v/v) Tween 20) with 5% non-fat dried milk for 1 h and incubated with appropriate primary antibodies overnight at 4°C. For visualization of primary antibodies, infrared (IR) dye conjugated secondary antibodies from LI-COR Biosciences were used: donkey α-mouse-IRdye680RD (1:2500, 926–68072), donkey α-mouse-IRdye800CW (1:2500, 926–32212), donkey α-rabbit-IRdye680RD (1:2500, 926–68073) and donkey α-rabbit-IRdye8000CW (1:2500, 926–32213). The fluorescence signals in the infrared range were detected using the Odyssey Infrared Imaging System from LI-COR Biosciences. Signal quantification was performed in Image Studio Lite software (LI-COR Biosciences). For each protein of interest, the average of the control/WT replicates was set at 100%, and experimental/S100A11-KO values were adjusted to this scale. Signal intensity values were quantified from at least three biological replicates.

### Plasma membrane wounding assays

#### Laser wounding assay

Cells were cultured on 8-well glass bottom μ-slides (ibidi), pre-coated with 50 μg/ml collagen (HUVEC only). At 60–80% confluency, cells were incubated in 2.5 mM CaCl_2_ or 100 μM EGTA containing Tyrode’s buffer (140 mg/ml NaCl, 5 mg/ml KCl, 1 mg/ml MgCl_2_, 10 mg/ml glucose, 10 mg/ml HEPES pH 7.4) supplemented with 5 μg/ml FM4-64 (Thermo Fisher Scientific, T13320). Live-cell imaging employed a Carl Zeiss LSM780 confocal microscope (heated to 37°C) with a Plan-Apochromat 63x/1.4 oil immersion objective. For all experiments, pinhole size was set to 1 Airy units and digital zoom was set to 1.3 with a scanning resolution of 1,220 × 1,220, resulting in a pixel size of 0.09 μm. To inflict wounds, a circular region of interest (ROI) with 20 pixels in diameter (2 μm^2^ surface area) was specified at flat extending membrane edges of cells. Laser ablation was performed with Chameleon Vision NLO laser (Coherent) at 820 nm wavelength with power set to 17% (corresponding to ∼4000 mW) for two iterations using bleaching mode in the ZEN software. A time series of 120 frames (each frame ∼1.56–1.89 s) was recorded, with the wounding taking place after the second frame. Time frames are reported as time points immediately before (pre-wounding) and after wounding with ablation occurring at t = 0. GFP/YFP or FM4-64 signals were acquired with a 488 nm laser and 525 ± 50 nm emission filter, while mApple signal was acquired with a 561 nm laser and 605 ± 70 nm emission filter.

#### Mechanical wounding assay

Mechanical wounding of EA.hy926 cells was performed as described for HUVEC using acid washed glass beads (425–600 μm; Sigma-Aldrich (G8772-100G)) and WT or S100A11-KO EA.hy926 cells cultured on 2-well glass bottom μ-slides (ibidi) (Supplementary Figure S3; Ashraf and Gerke, 2021).

### Immunoprecipitation

For immunoprecipitation (IP) experiments (all steps performed on ice), cell lysates were first prepared from EA.hy926 cells (60 mm culture dish) or HUVEC (100 mm dish). After washing in ice-cold PBS and sedimenting at 800 x g for 5 min, cells were lysed for 30 min in lysis buffer (250 mM sucrose, 20 mM HEPES pH 7.5, 0.5 mM EDTA pH 7.5 and complete Protease Inhibitor Cocktail) by passaging through a 21G x 1^1/2^ hollow needle at least thirty times for 10 min. The lysed mixtures were centrifuged at 14000 x g for 10 min at 4°C and the clear supernatants were saved as the lysates. IPs were conducted using GFP Selector beads (4% cross-linked agarose beads with covalently immobilized high-affinity single-domain antibodies (sdAb) which recognize GFP/YFP fusion proteins) from NanoTag Biotechnologies and proceeded with the ‘batch protocol’ steps as per the manufacturer with the following modifications: instead of using 20 µL of beads slurry per culture dish, we reduced the amount of beads slurry to 10 µL. For each condition, beads were equilibrated in wash buffer containing 250 mM sucrose, 20 mM HEPES pH 7.5, 0.5 mM EDTA pH 7.5, 2 mM CaCl_2_ and incubated with respective lysates for 1 h at 4°C under head-over-tail rotation. After binding, beads were washed thrice with the wash buffer. Input and the non-bound fractions were saved before and after the binding, respectively, and all samples were then incubated in 5x SDS sample buffer (boiled at 95°C for 5 min) and subjected to immunoblotting. IP and co-immunoprecipitation (Co-IP) efficiencies were calculated as the ratio of protein signal intensity in the beads sample multiplied with the value 0.02356 (percent of input loaded) against input fraction multiplied with the value 1 (percent of immunoprecipitated material loaded), represented as a percentage. As controls, non-transfected or cells transfected with empty GFP vector were used.

### Image analysis

All confocal microscopy images were analysed using Fiji (Schindelin et al., 2012). To measure changes in FM4-64 fluorescence intensity within the whole-cell over each time frame, a previously described macro (see Supplementary Material) utilizing “Plot Z-axis Profile” function was used (Ashraf et al., 2021). Plasma membrane wound repair efficiencies of laser-ablated cells were determined by generating time series plots of normalized FM4-64 fluorescence values (relative to pre-wounding). To capture wound-associated wave-like recruitment dynamics of fluorescently tagged S100A11 or annexin proteins, a circular ROI of radius 10 μm or 15 μm was defined around the wound site (for EA.hy926 cells and HUVEC, respectively). For each frame, fluorescence intensity changes of the respective fluorophore were measured within the area where the circular ROI overlaps the cell (Supplementary Figure S4). The values obtained were normalized to the initial fluorescence (relative to pre-wounding) after background correction and represented as a time series plot. The analysis steps were automated in a macro file which incorporated the “Plot Z-axis Profile” function (see Supplementary Material). To determine the repair efficiencies of mechanically wounded EA.hy926 cells, the numbers of FITC-Dextran stained cells (injured and repaired) and TRITC-Dextran stained cells (injured and non-repaired) were determined (post-thresholding FITC and TRITC channels) using “Cell Counter” plugin. Non-repaired cells were represented as a percentage of the total injured cells.

### Statistical analysis

For quantification purposes, experimental sets were repeated at least 3 times. Analysis results were grouped, arranged and measured in Microsoft Excel 2011. Throughout the paper, data are presented as mean ± SD. Statistical tests were performed in Prism 6 software (GraphPad). FM4-64 dynamics and wound-associated recruitment dynamics of proteins were analyzed based on area under the curve (AUC) values (threshold = 1.0) calculated for averages of each biological replicate. Statistical comparisons between groups were assessed with two-tailed Student’s t test or ordinary one-way ANOVA with Tukey’s multiple comparison test, as appropriate. Non-significant results were not specifically labeled. Asterisks mark statistically significant results: ****p ≤ 0.0001, ***p ≤ 0.001, **p ≤ 0.01, *p ≤ 0.05.

## Data Availability

The original contributions presented in the study are included in the article/Supplementary Material, further inquiries can be directed to the corresponding author.
