# Supplementary material for: The resealing factor S100A11 interacts with annexins and extended synaptotagmin-1 in the course of plasma membrane wound repair
Source: Front Cell Dev Biol. 2022 Sep 19;10:968164. doi: 10.3389/fcell.2022.968164 (PMC9527316; doi:10.3389/fcell.2022.968164)
Supplement: Supplementary file 5 [file DataSheet1.pdf]

Supplementary Material

Supplementary figures plus legends

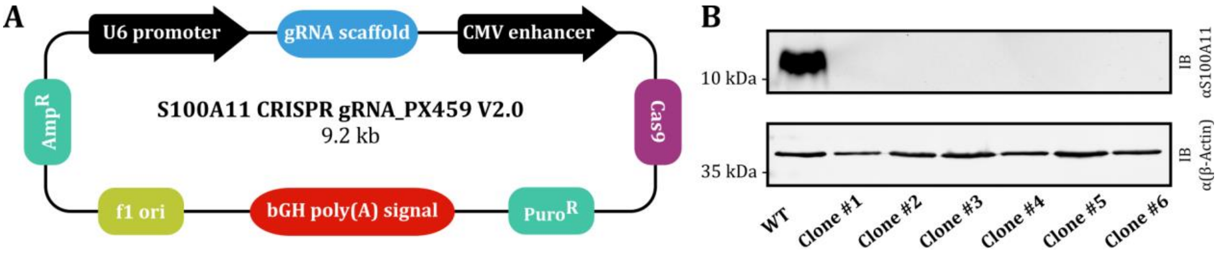

Figure S1. Generation of CRISPR/Cas S100A11 knockout cell lines. (A) Illustration of the S100A11 CRISPR gRNA _PX459 V2.0 plasmid to generate S100A11-KO EA.hy926 cells. **(B)** Immunoblot analysis of endogenous S100A11 (12 kDa) expression in WT and six different CRISPR/Cas S100A11-KO clones. β-actin (42 kDa) served as loading control. Representative blots from three independent experiments are shown.

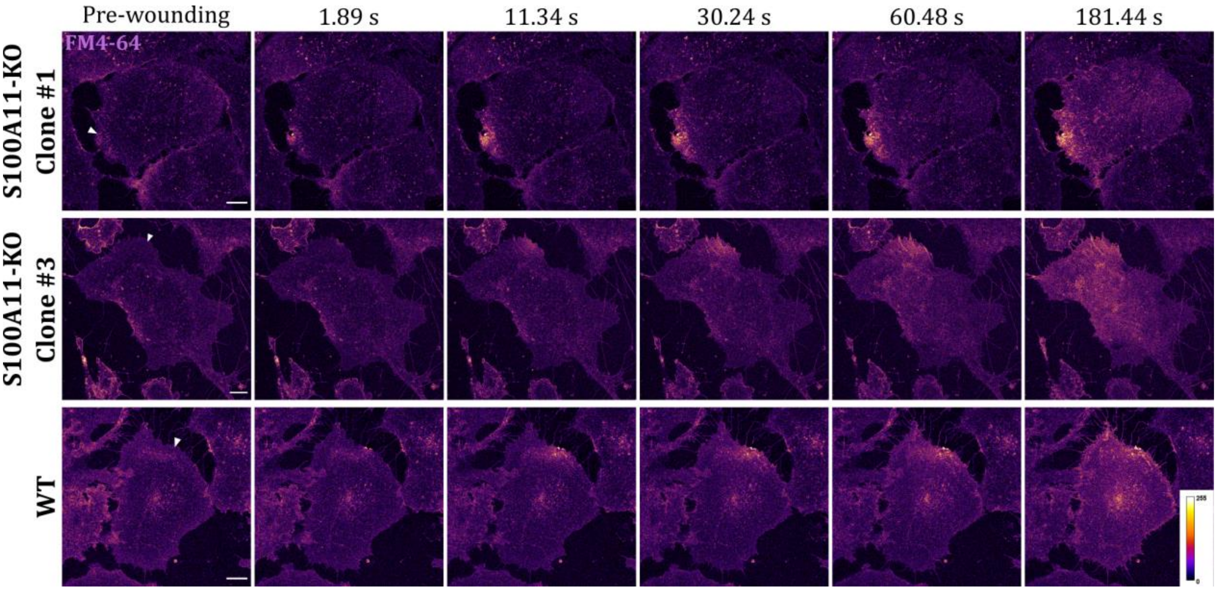

Figure S2. EA.hy926 cells repair plasma membrane wounds in a Ca^{2+}-dependent manner. Representative images of FM4-64 infiltration following membrane damage in S100A11-KO, clone #1 and #3, or WT EA.hy926 cells (Videos 2 and 4). Cells kept in Tyrode's buffer supplemented with 100 µM EGTA and 5 µg/ml FM4-64 were damaged with 820 nm light (near infrared) directed at the plasma membrane on the lateral edge (white triangles represent the wound sites). Wounding occurred at t = 0; pre-wounding and post-wounding time points are shown. Calibration bar of fluorescence intensity is provided on the lower right panel. Scale bars = 10 µm.

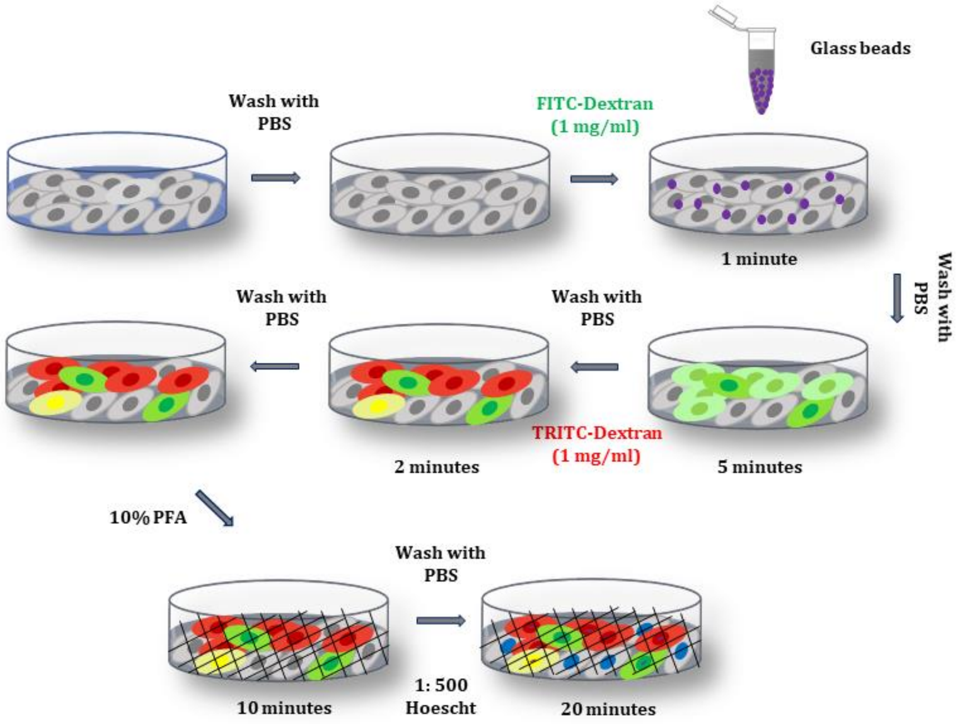

Figure S3. Mechanical wounding assay. Cells were wounded using glass beads in the presence of FITC-Dextran. Medium was then washed off and replaced with TRITC-Dextran containing medium. Subsequently, cells were fixed and stained for the cell nuclei using Hoechst. Injured and repaired cells are FITC-Dextran stained whereas cells unable to repair are stained in addition with TRITC-Dextran.

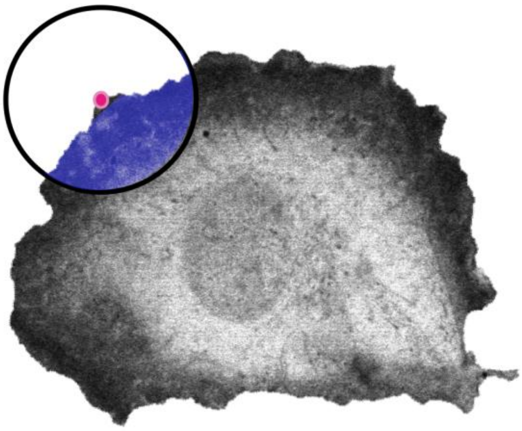

Figure S4. Quantification of wound-associated dynamics of fluorescently tagged Ca^{2+}-binding proteins. Illustration of circular ROI (black) defined around the wound site (red) for quantification. Circular ROIs with radius 10 µm and 15 µm were used for EA.hy926 cells and HUVEC, respectively. Fluorescence intensity changes of the respective fluorophore were measured within the area where the circular ROI overlaps the wounded cell.

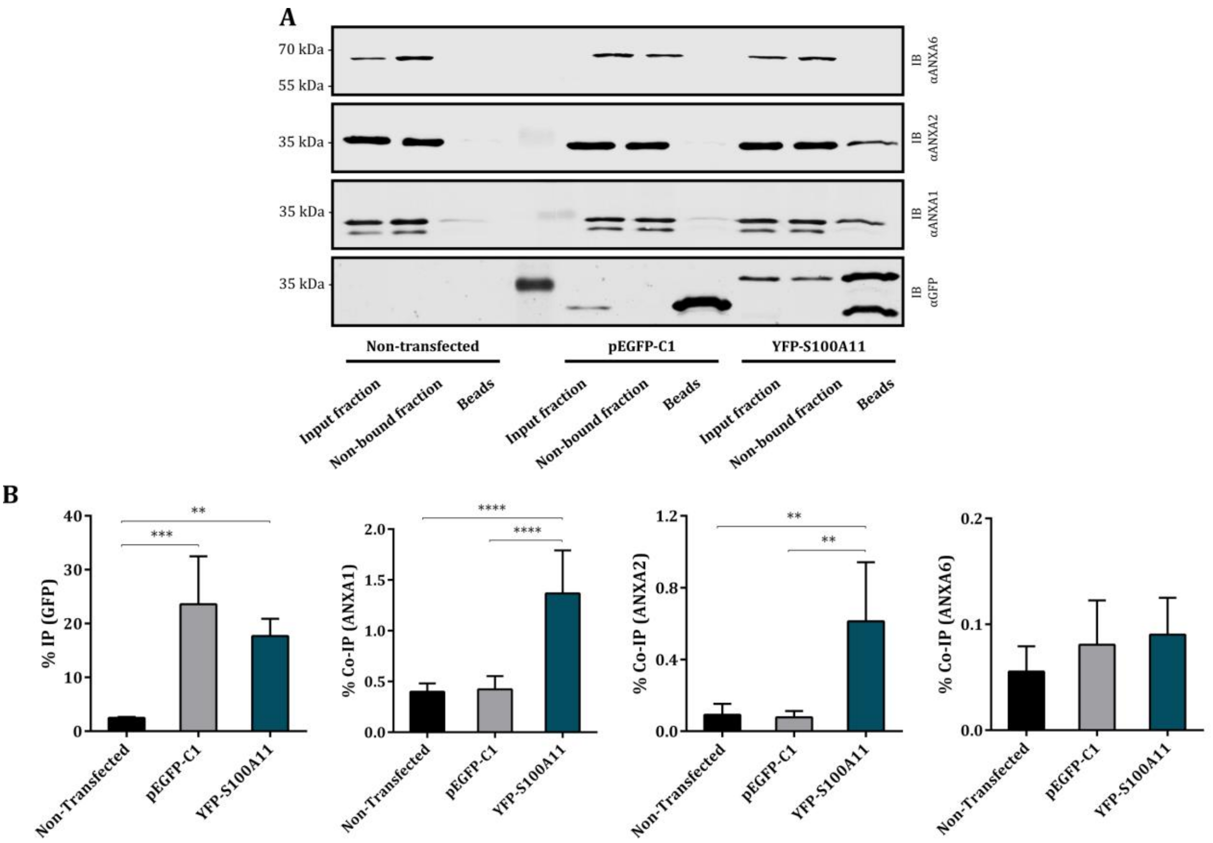

Figure S5. S100A11 interacts with ANXA1 and ANXA2 in endothelial cells. (A) Representative immunoblots of samples (input fraction, non-bound fraction and beads) from non-transfected HUVEC or HUVEC transfected with empty GFP vector or YFP-S100A11, which were subjected to IP reactions in the presence of 2 mM Ca^{2+}. Immunoblot analysis of immunoprecipitated empty GFP vector (27 kDa) or YFP-S100A11 (39 kDa) and co-immunoprecipitated endogenous ANXA1 (39 kDa), ANXA2 (38 kDa) or ANXA6 (68 kDa) are shown. Representative blots from five to nine independent experiments are shown. **(B)** IP efficiencies for empty GFP vector, YFP-S100A11 (left) and Co-IP efficiencies for endogenous ANXA1 (second graph from left), ANXA2 (second graph from right) or ANXA6 (right) quantified in five to nine independent blots. Statistical comparisons between groups were performed with ordinary one-way ANOVA (Tukey's multiple comparison test). Data are mean ± SD.

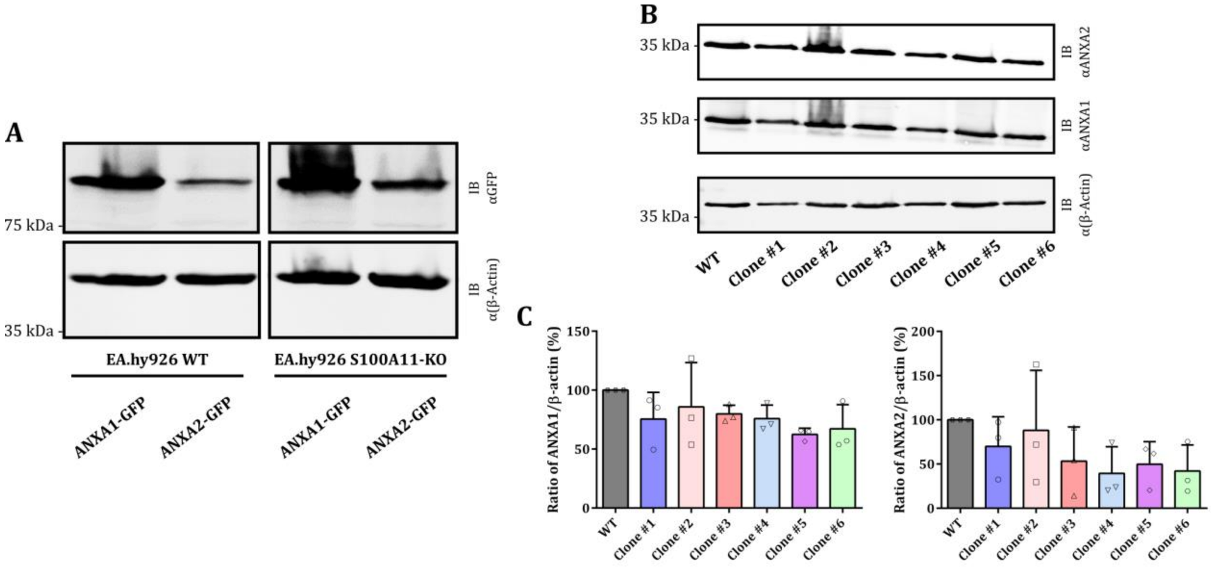

Figure S6. Expression of ANX constructs and endogenous ANX in WT and S100A11-KO cells. (A) Immunoblot analysis of ANXA1-GFP (66 kDa) or ANXA2-GFP (65 kDa) expression levels in WT (left) or S100A11-KO (right) cells transfected with respective constructs, individually. β-actin (42 kDa) served as loading control. **(B)** Immunoblot analysis of endogenous ANXA1 (39 kDa) or ANXA2 (38 kDa) expression in WT or six different S100A11-KO clones. β-actin (42 kDa) served as loading control. Representative blots from three independent experiments are shown. **(C)** Quantification of endogenous levels of ANXA1 (left) and ANXA2 (right) against β-actin, analyzed in three independent blots. Statistical comparisons between WT or S100A11-KO clones were performed with ordinary one-way ANOVA. Data are mean ± SD.

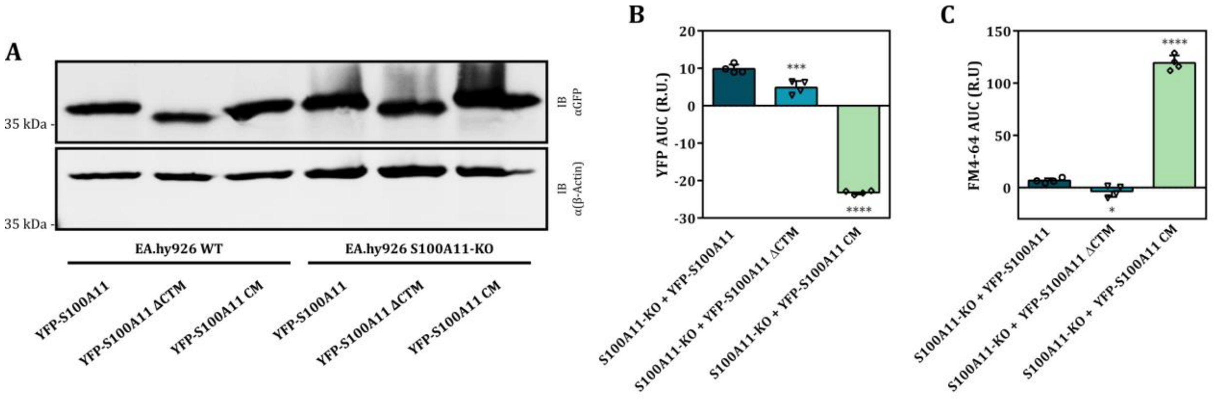

Figure S7. Expression and wound-associated dynamics of wild type S100A11, S100A11 ΔCTM and S100A11 CM in WT or S100A11-KO cells. (A) Immunoblot analysis of YFP-S100A11, YFP-S100A11 ΔCTM, YFP-S100A11 CM (~39 kDa) expression levels in WT or S100A11-KO cells transfected with respective constructs, individually. β-actin (42 kDa) served as loading control. **(B, C)** AUC values of YFP fluorescence (B) and FM4-64 fluorescence (C) for S100A11-KO cells transfected with YFP-S100A11, YFP-S100A11 ΔCTM or YFP-S100A11 CM and injured at the lateral membrane edge. Cells were kept in Tyrode's buffer supplemented with 2.5 mM Ca^{2+} and 5 µg/ml FM4-64. Results were pooled from four independent experiments (*n* = 24). Statistical analysis was performed with ordinary one-way ANOVA. Data are mean ± SD.

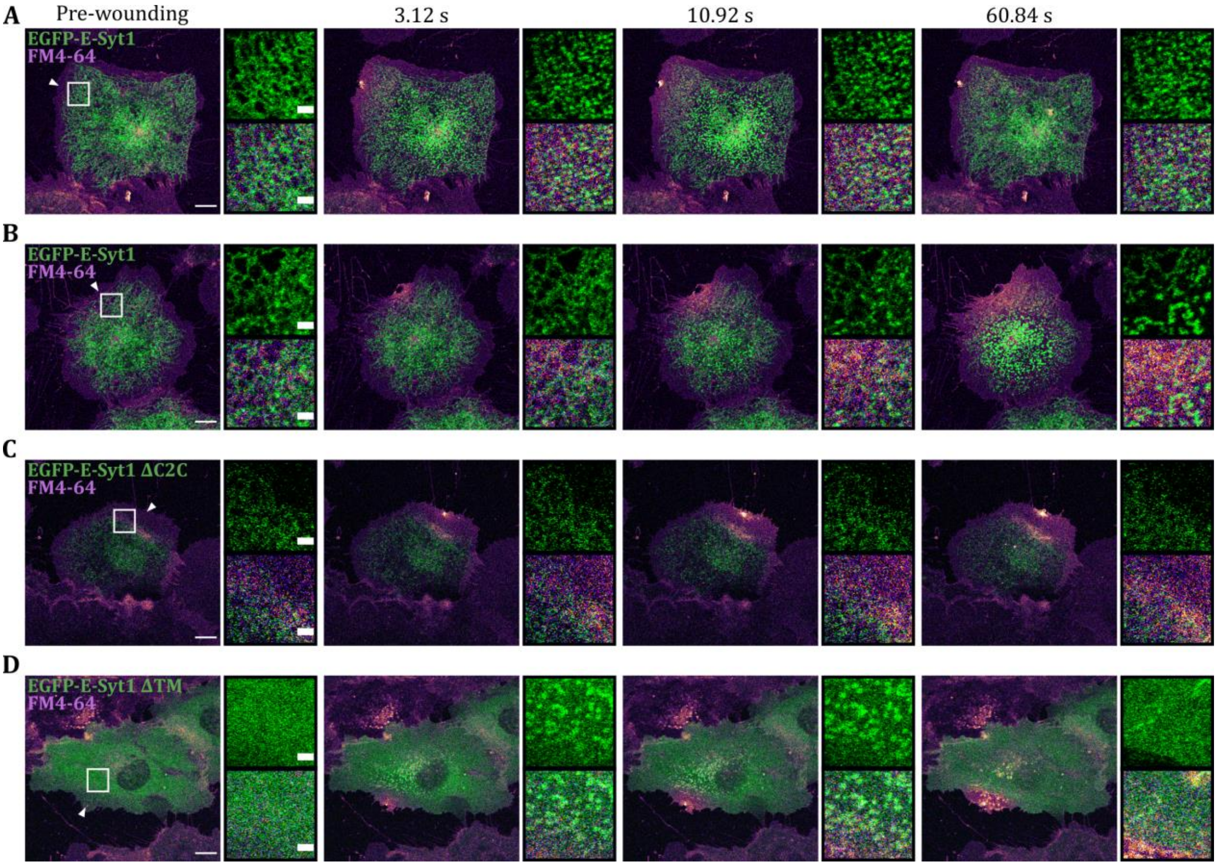

Figure S8. Plasma membrane wound-associated dynamics of wild type E-Syt1 as well as E-Syt1 ΔC2C and E-Syt1 ΔTM mutants in endothelial cells. (A, B) Time-lapse images of E-Syt1 dynamics in laser-ablated HUVEC. EGFP-E-Syt1 transfected cells kept in Tyrode's buffer supplemented with 2.5 mM Ca^{2+} (A) or 100 μM EGTA (B) and 5 μg/ml FM4-64 were injured at the lateral membrane edge (white triangles represent the wound sites) (Video 10). Wounding occurred at t = 0; pre-wounding and post-wounding time points are shown. Scale bars = 10 μm. Insets highlight the immediate vicinity around the wound. Scale bars = 2 μm. **(C, D)** Time-lapse images of E-Syt1 ΔC2C (C) or E-Syt1 ΔTM (D) dynamics in laser-ablated HUVEC. EGFP-E-Syt1 ΔC2C or EGFP-E-Syt1 ΔTM transfected cells kept in Tyrode's buffer supplemented with 2.5 mM Ca^{2+} and 5 μg/ml FM4-64 were injured at the lateral membrane edge (white triangles represent the wound sites). Control experiments carried out in the absence of extracellular Ca^{2+} (EGTA containing medium) showed no resealing under the experimental condition used here, indicative of a still Ca^{2+}-dependent repair process**.** Scale bars = 10 μm. Insets highlight the immediate vicinity around the wound. Scale bars = 2 μm.

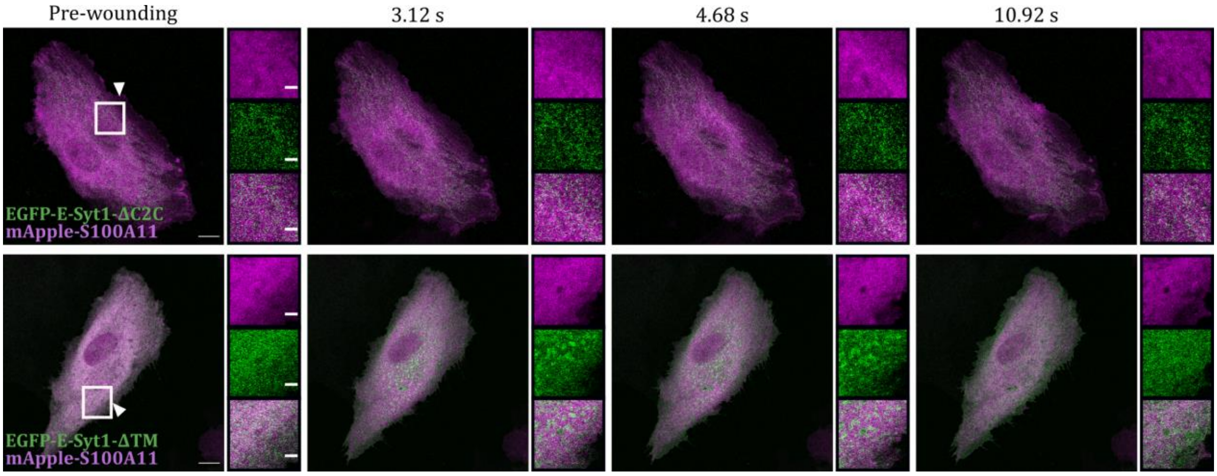

Figure S9. S100A11 recruitment dynamics in comparison to E-Syt1 ΔC2C and E-Syt1 ΔTM mutants during membrane wound repair in endothelial cells. Representative time-lapse images of injured HUVEC co-expressing EGFP-E-Syt1 ΔC2C and mApple-S100A11 (upper panel) or EGFP-E-Syt1 ΔTM and mApple-S100A11 (lower panel). Transfected cells kept in Tyrode's buffer supplemented with 2.5 mM Ca^{2+} were injured at the lateral membrane edge (white triangles represent the wound sites). Wounding occurred at t = 0; pre-wounding and post-wounding time points are shown. Scale bars = 10 µm. Insets highlight the immediate vicinity around the wound. Scale bars = 2 µm.

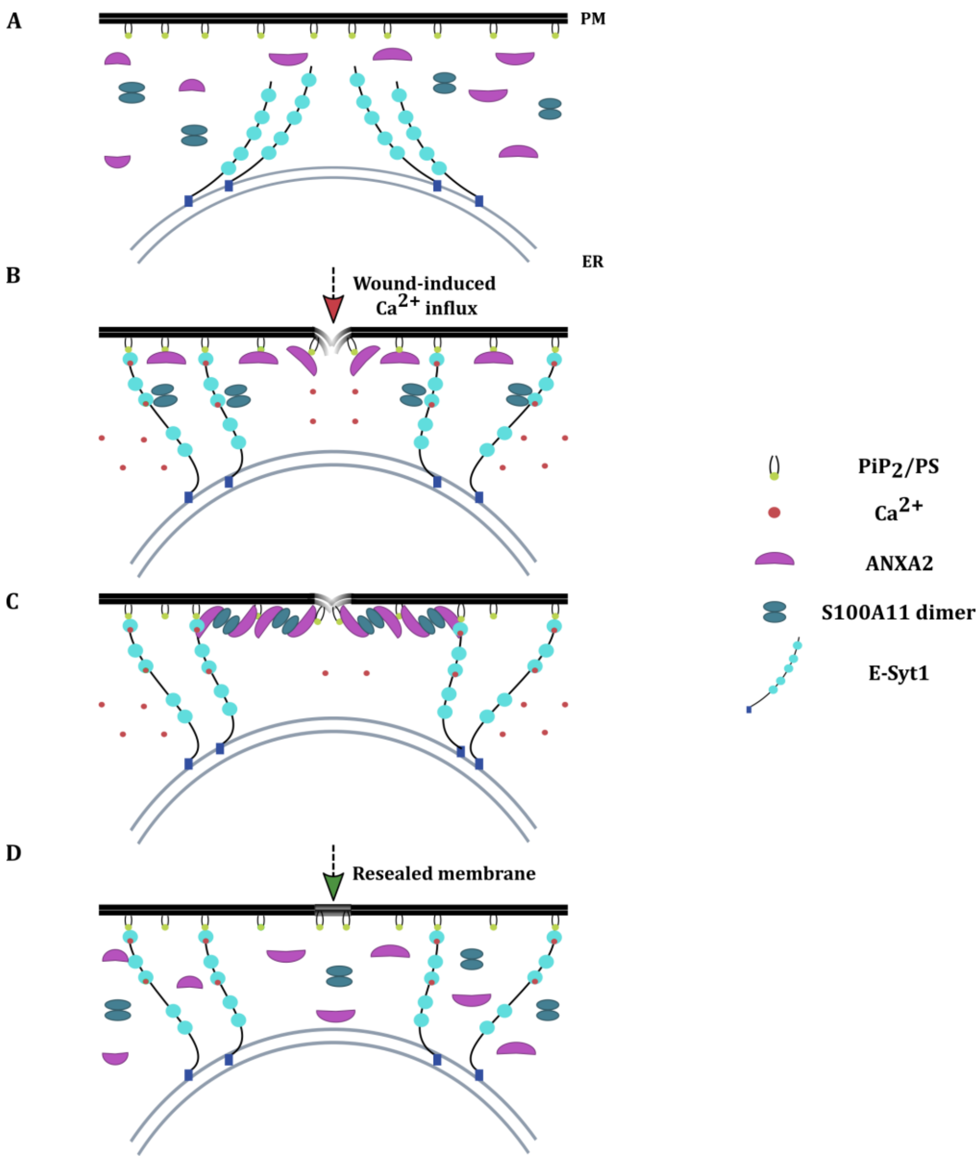

Figure S10. Simplified model depicting potential S100A11 interactions in the course of PM wound repair. Only the S100A11 interaction partners ANXA2 and E-Syt1 are shown. **(A)** In resting cells, ANXA2 and S100A11 homodimers are predominantly cytosolic, whereas E-Syt1 is localized to the ER membrane. **(B)** Cell injury leads to PM wound-associated Ca^{2+} influx, which triggers the formation of ER-PM contact sites rich in Ca^{2+}-bound E-Syt1 and the recruitment of Ca^{2+} bound S100A11 to such sites (via E-Syt1 binding). Moreover, Ca^{2+} induces the translocation of ANXA2 to the site of PM injury by Ca^{2+}-dependent binding to acidic phospholipids [such as PS and PI(4,5)P_2]. **(C)** Due to an increased local concentration of S100A11 at the PM, heterotetrameric S100A11-ANXA2 complexes form, possibly because of a somewhat higher S100A11 affinity of ANXA2 as compared to E-Syt1. These complexes are found enriched at and close to the wound site where the local ANXA2 concentrations are highest. At the actual wound site, the S100A11-ANXA2 heterotetramers, possibly in conjunction with other annexins and ANX-S100 complexes, could function in membrane repair by preferentially associating with the wound edges of the PM and supporting the formation of membrane bridges and/or a bending of the free membrane edges that eventually supports the actual resealing. **(D)** Following resealing of the PM and a reduction of cytosolic Ca^{2+} to resting levels, Ca^{2+}-free S100A11 will dissociate from ANXA2 and ANXA2 will be released from the membrane.

Video captions

Live-cell images were collected as described in the laser wounding assay section of Materials and Methods. Timestamps are provided in the top left corners. The playback rate is 2 frames per second. Scale bars = 10 μm.

Video 1 and 2. Plasma membrane wound repair is defective in S100A11-KO (clone 1) endothelial cells. The plasma membrane of S100A11-KO (clone 1) cells was ablated with 820 nm laser application in the presence of the lipophilic dye FM4-64 (red fire) and extracellular Ca^{2+} (Video 1) or EGTA (Video 2). Intracellular influx of the dye was monitored via time-lapse fluorescence confocal microscopy (white triangles represent the wound sites). Duration of each frame is 1.89 s.

Video 3 and 4. Plasma membrane wound repair in endothelial cells is Ca^{2+}-dependent. The plasma membrane of WT cells was ablated with 820 nm laser application in the presence of the lipophilic dye FM4-64 (red fire) and extracellular Ca^{2+} (Video 5) or EGTA (Video 6). Intracellular influx of the dye was monitored via time-lapse fluorescence confocal microscopy (white triangles represent the wound sites). Duration of each frame is 1.89 s.

Video 5. S100A11 translocates to the membrane wound site in S100A11-KO endothelial cells and rescues the membrane repair defect. A S100A11-KO cell transfected with a plasmid encoding YFP-S100A11 (cyan; left) was laser ablated in the presence of the lipophilic dye FM4-64 (red fire; right). Following membrane injury, recruitment of cytoplasmic S100A11 to the wound (left) was observed simultaneously with injury-induced FM4-64 influx (right) via time-lapse confocal fluorescence microscopy (white triangle represents the wound site). Duration of each frame is 1.56 s.

Video 6. ANXA1 translocates to the wound site in S100A11-KO endothelial cells and rescues the membrane repair defect. A S100A11-KO cell transfected with a plasmid encoding ANXA1-GFP (cyan; left) was laser ablated in the presence of the lipophilic dye FM4-64 (red fire; right). Following membrane injury, recruitment of ANXA1 to the wound (left) was observed simultaneously with injury-induced FM4-64 influx (right) via time-lapse confocal fluorescence microscopy (white triangle represents the wound site). Duration of each frame is 1.56 s.

Video 7. Absence of S100A11 disrupts membrane-wound associated translocation of ANXA2. A S100A11-KO cell transfected with a plasmid encoding ANXA2-GFP (cyan; left) was laser ablated in the presence of the lipophilic dye FM4-64 (red fire; right). Following membrane injury, recruitment dynamics of ANXA2 to the wound (left) was observed simultaneously with injury-induced FM4-64 influx (right) via time-lapse confocal fluorescence microscopy (white triangle represents the wound site). Duration of each frame is 1.56 s.

Video 8. S100A11 ΔCTM translocates to the wound site in S100A11-KO endothelial cells and rescues the membrane repair defect. A S100A11-KO cell transfected with a plasmid encoding YFP-S100A11 ΔCTM (cyan; left) was laser ablated in the presence of the lipophilic dye FM4-64 (red fire; right). Following membrane injury, recruitment of S100A11 ΔCTM to the wound (left) was observed simultaneously with injury-induced FM4-64 influx (right) via time-lapse confocal fluorescence microscopy (white triangle represents the wound site). Duration of each frame is 1.56 s.

Video 9. S100A11 CM fails to rescue the membrane wound repair defect in S100A11-KO endothelial cells. A S100A11-KO cell transfected with a plasmid encoding YFP-S100A11 CM (cyan; left) was laser ablated in the presence of the lipophilic dye FM4-64 (red fire; right). Following membrane injury, recruitment dynamics of S100A11 CM to the wound (left) was observed simultaneously with injury-induced FM4-64 influx (right) via time-lapse confocal fluorescence microscopy (white triangle represents the wound site). Duration of each frame is 1.56 s.

Video 10. ER-localized E-Syt1 translocates rapidly to punctate structures in the proximity of the membrane wound. HUVEC transfected with a plasmid encoding EGFP-E-Syt1 (green; left) was laser ablated in the presence of the lipophilic dye FM4-64 (red fire; right) and extracellular Ca^{2+}. Following membrane injury, wound-associated dynamics of EGFP-E-Syt1 (left) was observed simultaneously with injury-induced FM4-64 influx (right) via time-lapse confocal fluorescence microscopy (white triangle represents the wound site). Duration of each frame is 1.56 s.

Video 11. S100A11 colocalizes transiently with the Ca^{2+}-induced E-Syt1 punctate structures in the proximity of the membrane wound. HUVEC co-transfected with plasmids encoding EGFP-E-Syt1 (green; left) and mApple-S100A11 (magenta; right) was laser ablated. Following membrane injury, wound-associated dynamics of EGFP-E-Syt1 (left) was observed simultaneously with S100A11 recruitment (right) via time-lapse confocal fluorescence microscopy (white triangle represents the wound site). Duration of each frame is 1.56 s.

Video 12. Targeted knockdown of E-Syt1 and E-Syt2 attenuates the wound-directed recruitment of S100A11 in endothelial cells. HUVEC treated with E-Syt1 and E-Syt2-specific siRNAs and transfected with YFP-S100A11 (green; left) was laser ablated in the presence of the lipophilic dye FM4-64 (red fire; right). Following membrane injury, recruitment dynamics of S100A11 to the wound (left) were observed simultaneously with injury-induced FM4-64 influx (right) via time-lapse confocal fluorescence microscopy (white triangle represents the wound site). Duration of each frame is 1.56 s.

Video 13 and 14. Depletion of E-Syt1 and E-Syt2 interferes with proper membrane resealing in endothelial cells. HUVEC treated with control (Video 27) or E-Syt1 and E-Syt2-specific siRNAs (Video 28) were laser ablated in the presence of the lipophilic dye FM4-64 (red fire). Intracellular influx of the dye was monitored via time-lapse fluorescence confocal microscopy (white triangles represent the wound sites). Duration of each frame is 1.89 s.

Macro files for image analysis

Before running the macro files in Fiji, make sure that the folder contains only raw files. In the macro, channel numbers were specified based on the acquisition parameters of the time-lapse videos to be analyzed.

I. Quantification of FM4-64 influx dynamics in laser-ablated cells

As previously described (Ashraf et al., 2021), the following macro was designed to analyze the dynamics of FM4-64 dye in cells subjected to laser wounding. The macro allows to draw the outline of the cell of interest (COI) from the maximum-intensity projection of the acquired xyt image stack. For the outlined COI, the first frame of the FM4-64 channel was subtracted from the subsequent frames within the time series to remove background signals. Subsequently, the macro assessed fluorescence intensity changes using the "Plot Z-axis Profile" function. The processed fluorescence intensity values were exported into Microsoft Excel sheets.

```
run("Close All");
run("Options...", "iterations=1 count=1 black edm=Overwrite");

// Choose a directory that contains nothing but the source images
dir = getDirectory("Choose wisely...");

list = getFileList(dir);

resultPath = dir + File.separator + "Results"

File.makeDirectory(resultPath);

fileWithoutEnding = "";

for (i=0; i<list.length; i++) {
	if (isOpen("ROI Manager")) {
	selectWindow("ROI Manager");
	run("Close");
	}

	fileName = list[i];
	fileWithoutEnding = replace(fileName, "\\.[A-Za-z]+$", "");

	path = dir+fileName;

	if (!File.isDirectory(path)) {

		//Make maximum intensity projection of FM4-64 channel
		open(path);
		run("Duplicate...", "duplicate channels=1");
		run("Red");
		run("Z Project...", "projection=[Max Intensity]");
		maxprojredFile = "max proj red" + fileWithoutEnding + ".tif";
		saveAs("Tiff", resultPath + File.separator + maxprojredFile);

		//Draw ROIs by hand, add ROIs to ROI manager by pressing <t>, continue macro by pressing Enter,
		save ROIs as overlay
		run("ROI Manager...");
		waitForUser("draw ROIs by hand using the wand tool, add ROIs to ROI manager by pressing <t>,
		continue makro by clicking <OK>");
		run("From ROI Manager");
		ROIsFile = "ROIs" + fileWithoutEnding + ".tif";
		saveAs("Tiff", resultPath + File.separator + ROIsFile);
		selectWindow("ROI Manager");
		run("Close");
		run("Close All");
```

```
            //Make a file in which the first frame in FM4-64 channel is subtracted from each following frame
            open(path);
            run("Duplicate...", "duplicate channels=1");
            run("Red");
            redstackFile = "redstack " + fileWithoutEnding + ".tif";
            saveAs("Tiff", resultPath + File.separator + redstackFile);
            run("Duplicate...", " ");
            firstimgredFile = "first Img red " + fileWithoutEnding + ".tif";
            saveAs("Tiff", resultPath + File.separator + firstimgredFile);

            imageCalculator("Subtract create stack", redstackFile, firstimgredFile);
            resultredFile = "result red " + fileWithoutEnding + ".tif";
            saveAs("Tiff", resultPath + File.separator + resultredFile);
            run("Close All");

            //Plot the Z-Axis Profile for both ROIs, copy result to excel
            run("Plots...", "width=450 height=200 font=12 auto-close list minimum=0 maximum=0");
            open(resultPath + File.separator + ROIsFile);
            run("To ROI Manager");
            close("resultPath + File.separator + ROIsFile");
            open(resultPath + File.separator + resultredFile);
            selectWindow("ROI Manager");
            roiManager("Select", 0);
            run("Plot Z-axis Profile");
            selectWindow("Plot Values");
            ValuesCOIFile = resultPath + File.separator + "Values COI " + fileWithoutEnding + ".xls";
            run("Text...", "save=["+ ValuesCOIFile +"]");
        run("Close");
            selectWindow("ROI Manager");
            roiManager("Select", 1);
            run("Plot Z-axis Profile");
            selectWindow("Plot Values");
            ValuesBGFile = resultPath + File.separator + "Values BG " + fileWithoutEnding + ".xls";
            run("Text...", "save=["+ ValuesBGFile +"]");
        run("Close");
            selectWindow("ROI Manager");
            run("Close");
            run("Close All");

            print("Done with " + fileName);

        }
}

print("Done with all figures.");
```

II. Analysis of protein recruitment dynamics towards laser-induced membrane wounds in EA.hy926 cells

The macro below was designed to evaluate the wound-targeted recruitment of fluorescently tagged proteins of interest in laser-ablated EA.hy926 cells. Initially, the boundary of the cell was specified using the wand tool. For the outlined cell and the respective channel, the first frame was subtracted from the subsequent frames within the time series to remove background signals. Next, the macro defined a circular ROI of 10 µm radius around the wound ROI and calculated fluorescence intensity changes with the “Plot Z-axis Profile” function, in the area where the circular ROI overlaps the cell.

```
run("Close All");
run("Options...", "iterations=1 count=1 black edm=Overwrite");

// Choose a directory that contains nothing but the source images
dir = getDirectory("Choose wisely...");

list = getFileList(dir);
```

```
resultPath = dir + File.separator + "Results"
File.makeDirectory(resultPath);

fileWithoutEnding = "";

for (i=0; i<list.length; i++) {
		fileName = list[i];
		fileWithoutEnding = replace(fileName, "\\.[A-Za-z]+$", "");

		path = dir+fileName;

		if (File.isDirectory(path)) {
				continue;
		}

		resultPath = dir + File.separator +  "Results-" + fileWithoutEnding;
		File.makeDirectory(resultPath);

		//Make a file in which the first frame of respective channel is subtracted from each following frame
		open(path);
		run("Duplicate...", "duplicate channels=1");
		firststackFile = "firststack " + fileWithoutEnding + ".tif";
		saveAs("Tiff", resultPath + File.separator + firststackFile);
		run("Duplicate...", " ");
		firstimgfirstFile = "first Img first " + fileWithoutEnding + ".tif";
		saveAs("Tiff", resultPath + File.separator + firstimgfirstFile);

		imageCalculator("Subtract create stack", firststackFile, firstimgfirstFile);
		resultfirstFile = "result first " + fileWithoutEnding + ".tif";
		saveAs("Tiff", resultPath + File.separator + resultfirstFile);
		run("Close All");

		//Open image so that the ROIs are displayed in the ROI manager: the relevant box must be pre-clicked!
				open(path);
		//Make a saved overlay of the wound ROI, so that it is seen in the ROIsFile
		run("ROI Manager...");
		run("To ROI Manager");

		//Define the circular ROI around the wound
		roiManager("Select", 1);
		Roi.getBounds(x, y, width, height);
		rw = ((width / 2) - 1) * -1;
		run("Enlarge...", "enlarge=" + rw + " pixel");
		run("Enlarge...", "enlarge=10");
		roiManager("Add");

		//Draw the boundary of the cell of interest
		waitForUser("draw COI by hand using the wand tool, add to ROI manager by pressing <t>, continue macro by
		clicking <OK>");
		run("From ROI Manager");

		//all ROIs specified above and the wounding ROI will be saved as an overlay in the ROIs file
		ROIsFile = "ROIs" + fileWithoutEnding + ".tif";
		saveAs("Tiff", resultPath + File.separator + ROIsFile);
		run("Close All");

		//Make Plot Z axis profile of the region
		open(resultPath + File.separator + resultfirstFile);
		roiManager("Select", 2);
		run("Clear Outside", "stack");
		Plot1File = "Plot1 " + fileWithoutEnding + ".tif";
		saveAs("Tiff", resultPath + File.separator + Plot1File);
		Plot1File = "Plot1" + fileWithoutEnding + ".tif";
		saveAs ("Tiff", resultPath + File.separator + Plot1File);
		run("Plot Z-axis Profile");
		ImagePlot1File = "Plot1" + fileWithoutEnding + ".tif";
```

```
		saveAs("Tiff", resultPath + File.separator + ImagePlot1File);
		run("Close");

		selectWindow("ROI Manager");
		run("Close");

		run ("Close All");

		print("Done with " + fileName);

		run("Close All");
}

print("Done with all pictures.");
```

III. Analysis of protein recruitment dynamics towards laser-induced membrane wounds in HUVEC

The macro below is similar to (B), but defined a circular ROI of 15 µm radius around the wound ROI to capture the wound-associated recruitment of fluorescently tagged proteins of interest in laser-ablated HUVEC.

```
run("Close All");
run("Options...", "iterations=1 count=1 black edm=Overwrite");

// Choose a directory that contains nothing but the source images
dir = getDirectory("Choose wisely...");

list = getFileList(dir);

resultPath = dir + File.separator + "Results"
File.makeDirectory(resultPath);

fileWithoutEnding = "";

for (i=0; i<list.length; i++) {
		fileName = list[i];
		fileWithoutEnding = replace(fileName, "\\.[A-Za-z]+$", "");

		path = dir+fileName;

		if (File.isDirectory(path)) {
				continue;
		}

		resultPath = dir + File.separator +  "Results-" + fileWithoutEnding;
		File.makeDirectory(resultPath);

		//Make a file in which the first frame of respective channel is subtracted from each following frame
		open(path);
		run("Duplicate...", "duplicate channels=1");
		firststackFile = "firststack " + fileWithoutEnding + ".tif";
		saveAs("Tiff", resultPath + File.separator + firststackFile);
		run("Duplicate...", " ");
		firstimgfirstFile = "first Img first " + fileWithoutEnding + ".tif";
		saveAs("Tiff", resultPath + File.separator + firstimgfirstFile);

		imageCalculator("Subtract create stack", firststackFile, firstimgfirstFile);
		resultfirstFile = "result first " + fileWithoutEnding + ".tif";
		saveAs("Tiff", resultPath + File.separator + resultfirstFile);
		run("Close All");

		//Open image so that the ROIs are displayed in the ROI manager: the relevant box must be pre-clicked!
				open(path);
		//Make a saved overlay of the wound ROI, so that it is seen in the ROIsFile
		run("ROI Manager...");
```

```
        run("To ROI Manager");

        //Define the circular ROI around the wound
        roiManager("Select", 1);
        Roi.getBounds(x, y, width, height);
        rw = ((width / 2) - 1) * -1;
        run("Enlarge...", "enlarge=" + rw + " pixel");
        run("Enlarge...", "enlarge=10");
        roiManager("Add");

        //Draw the boundary of the cell of interest
        waitForUser("draw COI by hand using the wand tool, add to ROI manager by pressing <t>, continue macro by
        clicking <OK>");
        run("From ROI Manager");

        //all ROIs specified above and the wounding ROI will be saved as an overlay in the ROIs file
        ROIsFile = "ROIs" + fileWithoutEnding + ".tif";
        saveAs("Tiff", resultPath + File.separator + ROIsFile);
        run("Close All");

        //Make Plot Z axis profile of the region
        open(resultPath + File.separator + resultfirstFile);
        roiManager("Select", 2);
        run("Clear Outside", "stack");
        Plot1File = "Plot1 " + fileWithoutEnding + ".tif";
        saveAs("Tiff", resultPath + File.separator + Plot1File);
        Plot1File = "Plot1" + fileWithoutEnding + ".tif";
        saveAs ("Tiff", resultPath + File.separator + Plot1File);
        run("Plot Z-axis Profile");
        ImagePlot1File = "Plot1" + fileWithoutEnding + ".tif";
        saveAs("Tiff", resultPath + File.separator + ImagePlot1File);
        run("Close");

        selectWindow("ROI Manager");
        run("Close");

        run ("Close All");

        print("Done with " + fileName);

        run("Close All");
}

print("Done with all pictures.");
```
